# New perspectives on an old grouping: The genomic and phenotypic variability of *Oxalobacter formigenes* and the implications for calcium oxalate stone prevention

**DOI:** 10.3389/fmicb.2022.1011102

**Published:** 2022-12-21

**Authors:** John A. Chmiel, Charles Carr, Gerrit A. Stuivenberg, Robertson Venema, Ryan M. Chanyi, Kait F. Al, Daniel Giguere, Henry Say, Polycronis P. Akouris, Sergio Ari Domínguez Romero, Aaron Kwong, Vera Tai, Susan F. Koval, Hassan Razvi, Jennifer Bjazevic, Jeremy P. Burton

**Affiliations:** ^1^Department of Microbiology and Immunology, Western University, London, ON, Canada; ^2^Canadian Centre for Human Microbiome and Probiotics Research, London, ON, Canada; ^3^Department of Molecular Genetics, University of Toronto, Toronto, ON, Canada; ^4^Department of Medicine, The University of British Columbia, Vancouver, BC, Canada; ^5^School of Veterinary Science, Massey University, Palmerston North, New Zealand; ^6^Department of Biology, Western University, London, ON, Canada; ^7^Department of Medicine, Western University, London, ON, Canada; ^8^Division of Urology, Department of Surgery, Western University, London, ON, Canada

**Keywords:** oxalate degradation, *Oxalobacter formigenes*, kidney stone disease, gut microbiome, revised taxonomy, phylogenomic and comparative genomic analyses, nephrolithiasis and hyperoxaluria

## Abstract

*Oxalobacter formigenes* is a unique bacterium with the ability to metabolize oxalate as a primary carbon source. Most kidney stones in humans are composed of calcium and oxalate. Therefore, supplementation with an oxalate-degrading bacterium may reduce stone burden in patients suffering from recurrent calcium oxalate-based urolithiasis. Strains of *O. formigenes* are divided into two groups: group I and group II. However, the differences between strains from each group remain unclear and elucidating these distinctions will provide a better understanding of their physiology and potential clinical applications. Here, genomes from multiple *O. formigenes* strains underwent whole genome sequencing followed by phylogenetic and functional analyses. Genetic differences suggest that the *O. formigenes* taxon should be divided into an additional three species: *Oxalobacter aliiformigenes* sp. nov, *Oxalobacter paeniformigenes* sp. nov, and *Oxalobacter paraformigenes* sp. nov. Despite the similarities in the oxalyl-CoA gene (*oxc*), which is essential for oxalate degradation, these strains have multiple unique genetic features that may be potential exploited for clinical use. Further investigation into the growth of these strains in a simulated fecal environment revealed that *O. aliiformigenes* strains are capable of thriving within the human gut microbiota. *O. aliiformigenes* may be a better therapeutic candidate than current group I strains (retaining the name *O. formigenes*), which have been previously tested and shown to be ineffective as an oral supplement to mitigate stone disease. By performing genomic analyses and identifying these novel characteristics, *Oxalobacter* strains better suited to mitigation of calcium oxalate-based urolithiasis may be identified in the future.

## Introduction

The global prevalence of kidney stones ranges from 0.2% to 20% (Romero et al., 2010; Turney et al., 2012; Wang et al., 2017) and nearly 10% of North Americans have experienced a kidney stone during their lifetime (Scales et al., 2012). Furthermore, the global prevalence of this disease has been steadily increasing and is a major financial burden to healthcare systems worldwide (Romero et al., 2010). Stones develop in the kidneys and can become lodged in the urinary system where they can obstruct the flow of urine and potentially cause debilitating pain, infection, and renal dysfunction. While many different types of kidney stones exist, approximately 80% contain calcium oxalate (Singh et al., 2015).

Urinary oxalate originates from both dietary and endogenous sources. Some foods that are high in oxalate include strawberries, spinach and other dark leafy green vegetables, chocolate, nuts, and wheat bran (Massey et al., 1993). Oxalate is also a normal metabolic by-product of hepatic glycolate metabolism. Glycolate is converted into oxalate through a glyoxylate intermediate and enters into systemic circulation (Baker et al., 2004). Individuals with primary hyperoxaluria have a defect in glycolate metabolism, which increases oxalate production. Although oxalate is an end product of human metabolism, humans do not possess the enzymes to metabolize oxalate (Daniel et al., 2021). Instead, the molecule is either released into the intestinal lumen for fecal excretion or the renal system for urinary excretion (Whittamore and Hatch, 2017).

The gut microbiota includes a diverse consortium of bacteria capable of metabolizing oxalate (Liu et al., 2021). Notably, *Oxalobacter formigenes* (Allison et al., 1985) is a Gram-negative, anaerobic bacterium that can utilize oxalate as its primary carbon source. First isolated from the rumen of a sheep in the 1980s (Dawson et al., 1980), *O. formigenes* has now been identified in the gastrointestinal tract of various mammals, including humans, and freshwater lake sediment (Smith et al., 1985). In addition to consuming oxalate, this bacterium also requires acetate but cannot use it as a sole carbon source (Cornick and Allison, 1996). There have been multiple attempts to characterize isolates of *O. formigenes* to better understand the physiology and functional applications of this organism. Based on cellular fatty acid composition, *O. formigenes* was first separated into two distinct groups: group I and group II (Allison et al., 1985; Garrity et al., 2005). This separation was largely corroborated by analysis of 16S rRNA gene sequences and DNA probes specific for *oxc* (oxalyl-CoA decarboxylase) and *frc* (formyl-CoA transferase), but there are still areas lacking clarity with certain strains poorly classified (Sidhu et al., 1997; Garrity et al., 2005). Within the groupings, there are differences in antibiotic resistance, survival in bile salts, viability in low pH, tolerance to oxygen, and cultivability (Duncan et al., 2002; Lange et al., 2012). Taken together, these physiological differences could be the result of increased genetic diversity between group I and group II strains that has not been previously characterized.

Due to its ability to consume free oxalate as a carbon source, the presence of *O. formigenes* in the gastrointestinal tract has been speculated to lower the risk of calcium oxalate kidney stones. While some studies have demonstrated a negative correlation between *O. formigenes* abundance and oxalate-based stones (Kaufman et al., 2008), more recent findings appear to indicate the contrary (Tang et al., 2018; Ticinesi et al., 2018). To mitigate primary hyperoxaluria, attempts have been made to supplement individuals with group I strains, which are more prevalent in the human intestinal microbiome (Duncan et al., 2002; Barnett et al., 2016; Liu et al., 2017a). These clinical trials were largely unsuccessful in achieving colonization or reducing urinary oxalate excretion (Hoppe et al., 2006, 2017; Milliner et al., 2018). However, the clinical potential of group II strains still remains unknown.

Despite the classification system for *O. formigenes*, the differences between group I and group II strains have not been well established. The current methods are limited in identifying diversity, thus narrowing their conclusions. Given the unique metabolism of *O. formigenes* and the poorly described functional differences between group I and group II strains, we set out to classify and characterize multiple *O. formigenes* strains on a genetic level. Additionally, we performed *in vitro* analyses to determine how the growth of *Oxalobacter* spp. could be modulated through exogenous oxalate or individual strain supplementation. These investigations advance the understanding of these strains’ physiology, role in oxalate degradation, and potential for human therapeutic applications.

## Materials and methods

### Bacterial strains and cultures

*Oxalobacter formigenes* strains are described in Supplementary Table S1 and were routinely cultured in Hungate tubes as described by Hungate (1950). Strains were grown in anaerobic oxalate broth with various amounts of oxalate added at 37°C for up to 3 days (Daniel et al., 2021). The broth was modified by increasing the acetate concentration to 5 mM. Twenty millimolar oxalate was used for initial culturing from glycerol freezer stocks and 50 mM was used for increased propagation of the bacteria.

### Genomic sequencing and assembly

*Oxalobacter formigenes* strains BA1, OxGP1, and OxB^T^ were sequenced using short-read sequencing techniques. Briefly, cells were grown in oxalate broth media and DNA was extracted using a PureLink® Genomic DNA Mini Kit (Invitrogen, Burlington, ON) as per the manufacturer’s instructions. Quality and quantity of DNA was confirmed using a Nanodrop ND-1000 spectrophotometer (Thermo Fisher Scientific) and Qubit 2.0 fluorometer (Life Technologies). Genomic libraries were prepared using a Nextera XT kit (Illumina Technologies, USA) and sequencing was performed using a MiSeq (Illumina Technologies, USA) at the London Regional Genomics Center at the Robarts Research Institute (London, Canada).

The quality of 2 × 150 bp paired-end reads was assessed using FastQC v0.11.9 (Andrews, 2010). Reads were trimmed and filtered using fastp v0.23.0 (Chen et al., 2018). The average read quality was set to 27, --cut_front and --cut_tail flags were added with a mean quality of 30, and the minimum length was set to 35 bp. Short reads were assembled using SPAdes v3.15.3 (Bankevich et al., 2012) optimized with the Unicycler v0.5.0 (Wick et al., 2017) platform using normal mode.

Strains sequenced using long-read sequencing technologies were grown for up to 3 days in oxalate broth media containing 100 mM of oxalate. Genomic DNA was extracted using a slightly modified phenol-chloroform method. In brief, bacterial cultures were lysed for 2 hours at 37°C in lysis buffer (100 mM NaCl, 10 mM Tris–HCl (pH 8.0), 25 mM EDTA (pH 8.0), 0.5% (w/v) SDS, 100 μg/ml lysozyme, and 100 μg/ml RNAse A). Protein degradation was performed by adding Proteinase K (100 μg/ml final concentration) and incubating at 50°C for 2 hours. Nucleic acid content was purified using two rounds of 1 volume of 25:24:1 phenol:chloroform:isoamyl alcohol and washed twice with 1 volume of chloroform. Next, 0.1 volumes of 3 M sodium acetate (pH 5.2) were added to the aqueous phase and 2 volumes of 100% ice-cold ethanol were added to promote DNA precipitation. The DNA was pelleted by centrifuging at 4,500 × *g* for 10 min (room temperature) and washed twice with 70% ethanol. The purified DNA was resuspended in 10 mM Tris–HCl (pH 9.0). Quality, quantity, and DNA size were confirmed using a Nanodrop ND-1000 spectrophotometer (Thermo Fisher Scientific), Qubit 2.0 fluorometer (Life Technologies), and gel electrophoresis, respectively. The sequencing library was prepared from the genomic DNA using Oxford Nanopore’s ligation sequencing kit (SQK-LSK109) with its native barcoding expansion kit (EXP-NBD104). Sequencing was performed on an Oxford Nanopore MinION R9.4.1 flow cell (FLO-MIN106D) and basecalling was performed using Guppy v5.0.16 in high accuracy mode. Basecalled reads were filtered and trimmed with NanoFilt v2.7.1 (De Coster et al., 2018). Initial assemblies were constructed using Flye v2.8.3 (Kolmogorov et al., 2019), then filtered reads were mapped against draft assemblies with Minimap2 v2.17 (Li, 2018) and subsequently polished with Racon v1.4.13 (Vaser et al., 2017) and Medaka v1.4.3.

### Phylogenetic analysis and comparative genomics

All available *O. formigenes* genome assemblies were downloaded from NCBI (December 2021). All genomes (including our genome assemblies) were assessed for quality using QUAST v5.0.2 (Gurevich et al., 2013) and completeness using CheckM v1.1.3 (Parks et al., 2015). Genome assemblies with N50 < 10 kb, completeness of less than 95%, and contamination greater than 5% were excluded from further analysis. An exception was made for *O. formigenes* HOxHM18, which was sequenced in our study and produced an assembly with completion of 87.3%, contamination of 1.63% and N50 of 2.28 Mb. A total of 22 genome assemblies passed these quality control thresholds. All strains were annotated using the Prokka v1.14.6 pipeline (Seemann, 2014), which uses Prodigal v2.6.3 (Hyatt et al., 2010) for gene prediction. Orthologous protein sequences were determined using OrthoFinder v2.5.4 (Emms and Kelly, 2019) in blast mode. Single copy core ortholog sequences were aligned using MUSCLE v5.1 (Edgar, 2021), trimmed using trimAl v1.4.1 (Capella-Gutiérrez et al., 2009) using the -automated1 flag, and concatenated into a new alignment. A maximum likelihood phylogenetic tree was constructed using the concatenated single-copy core ortholog sequences in RAxML v8.2.12 (Stamatakis, 2014) with the flags -f a, -# autoMRE, and -m PROTGAMMAAUTO (LG was selected as the best substitution model), and *Burkholderia cepacia* ATCC 25416^T^ (GCA_003546465.1) was used as the outgroup. The tree was visualized using the R package ggtree v3.2.1 (Yu et al., 2017). Average nucleotide identity was calculated with FastANI v 1.32 (Jain et al., 2018), average amino acid identity was calculated using CompareM v0.1.2, digital DNA–DNA hybridization was determined with the DSMZ online tool GGDC v3.0^1^ using the default settings and the values from Formula 2 (Meier-Kolthoff et al., 2022). Matrices were visualized with the ComplexHeatmap v2.10.0 (Gu et al., 2016) wrapper for pheatmap v1.0.12 (Kolde, 2019). The pangenome was constructed using Roary v3.13.0 with the assumptions that a core gene is defined as a gene found in all but one of the isolates (>95%) and a minimum percentage identify for blastp of 90% (Page et al., 2015). Related gene content was further analyzed using the R package UpSetR v1.4.0 (Conway et al., 2017).

### 16S rRNA gene alignment

All available *O. formigenes* 16S rRNA gene sequences that were from strains not present in this study were downloaded from NCBI (January 2022). Note, the *O. formigenes* HRGM_Genome_1325 was excluded because the 16S rRNA gene sequence was not annotated by the Prokka pipeline. The 16S rRNA gene from the *B. cepacia* ATCC 25416^T^ genome assembly (GCA_003546465.1) was also acquired. Gene sequences were aligned with MAFFT v7.490 (Katoh and Standley, 2013) with the --auto flag. A maximum likelihood phylogenetic tree was constructed on the 16S rRNA gene sequence alignment using RAxML v8.2.12 (Stamatakis, 2014), with the flags -f a, -# autoMRE, and -m GTRGAMMA, and the 16S rRNA gene sequence of *B. cepacia* ATCC 25416^T^ was used as the outgroup. Majority-based consensus sequences for each *Oxalobacter* species were determined using the web-based EMBOSS program cons. The phylogenetic tree and gene sequence alignment was visualized using ggtree v3.2.1 (Yu et al., 2017), NCBI Multiple Sequence Alignment viewer v1.21.0, and ggmsa v1.0.0 (Zhou and Yu, 2021).

### Genome annotation and functional analysis prediction

Coding sequences were further annotated using eggNOG v5.0 and web-based eggNOG-mapper v2^2^ using the auto taxonomic scope (Huerta-Cepas et al., 2017, 2019). A Bray–Curtis dissimilarity distance matrix for functional category abundance were calculated from absolute abundances of clusters of orthologous groups (COG) categories and a PCoA was performed using the R package vegan v2.5–7 (Oksanen, et al., 2020) following an established method (Wuyts et al., 2017), and plotted using the R package ggplot2 v3.3.5 (Wickham, 2016). PERMANOVA (adonis2) was used to test for significant differences in COG category abundances between species (R package vegan v2.5–7; Oksanen, et al., 2020) and pairwise comparisons were made with the wrapper pairwiseAdonis (Martinez Arbizu, 2020). Abundances of each COG were visualized with the ComplexHeatmap v2.10.0 (Gu et al., 2016) wrapper for pheatmap v1.0.12 (Kolde, 2019). Cas clusters were identified using the web-based CRISPRCasFinder^3^ (Couvin et al., 2018). Bacteriocins were annotated by uploading the genomes to the BAGEL4 web server^4^ (van Heel et al., 2018, p. 4). Antibiotic resistance genes were annotated using AMRFinderPlus v3.10.20 (Feldgarden et al., 2021). Prophages were identified using the PHASTER web server^5^ (Zhou et al., 2011; Arndt et al., 2016). Cas genes were visualized using gggenes v0.4.1 (Wilkins, 2020), bacteriocins and antibiotic resistance genes were visualized using circlize v0.4.13 (Gu et al., 2014), and prophages were visualized with ggplot2 v3.3.5 (Wickham, 2016).

### *In silico* oxalyl-CoA decarboxylase analysis

All amino acid sequences of the oxalyl-CoA decarboxylase protein were collected from the genomes and were aligned using MUSCLE v5.1 (Edgar, 2021). Species-specific consensus sequences were determined using the web-based EMBOSS program cons^6^ and aligned using MUSCLE v5.1 (Edgar, 2021) before viewing on the NCBI Multiple Sequence Alignment viewer v1.21.0^7^.

To predict the protein structure of oxalyl-CoA decarboxylase produced by various *Oxalobacter* spp., the individual amino acid sequences were uploaded to the SWISS-MODEL server in FASTA format. To select an appropriate template for modeling, the uploaded sequences served as a query to identify an evolutionarily related protein by scanning both the BLAST (Camacho et al., 2009) and HHblits (Steinegger et al., 2019) databases. Of the ~1,500 proteins assessed, PDB entry 2IJ7 (Berthold et al., 2007) was chosen as the template because it was predicted to yield the highest quality models as estimated by Global Model Quality Estimate (GMQE; Biasini et al., 2014) and Quaternary Structure Quality Estimate (QSQE; Bertoni et al., 2017). Using this template, SWISS-MODEL generated individual 3D models by leveraging the OpenStructure computational structural biology framework and the ProMod3 modeling engine (Studer et al., 2020); a more detailed explanation of the model building process is available elsewhere (Waterhouse et al., 2018). The accuracy and quality of the predicted structures were determined sufficient according to an accepted scoring system (QMEAN Z-score of ~0; Benkert et al., 2011). The affinity of the oxalyl-CoA decarboxylase models to the natural ligand, oxalyl-CoA (ChEBI ID: CHEBI:15535), was then predicted. Using AutoDock Vina v1.2.0 (Trott and Olson, 2010), the ligand was targeted to protein complexes using residues Arg408 or Arg409 to define the binding center, and docking calculations were carried out with default parameters. The docking poses with the best docking score (predicted affinity) were selected for further analysis and were imaged using Open-Source pyMOL v2.5.0.

### Collection and preparation of fecal inoculums

The study was approved by the Health Sciences Research Ethics Board at the University of Western Ontario (REB #119537). Two healthy male donors provided fresh stool samples. None of the donors had a recent history (within 30 days) of antibiotic exposure of donation. Fecal samples were processed as follows and were stored at-80°C until required.

### Simulated model of the human gut microbiota

The effect of oxalate exposure on the human gut bacterial community and *Oxalobacter* was evaluated in a simulated human gut microbiota model of the distal colon using a Bioflo 110 bioreactor (New Brunswick Scientific, Edison, NJ). The design is improved from previous iterations (McDonald et al., 2013), with the addition of an air break between the media pump and chemostat vessel, constructed from three fused 10 ml syringes, to minimize contamination. Growth medium was formulated as previously described and was replenished at a rate of 16 ml/h (Daisley et al., 2020). The chemostat was maintained in an anaerobic state by continual gassing with nitrogen. The chemostat contents were maintained at 37°C and agitated at 100 RPM. A pH of 7.0 was maintained by the continuous flow of 1 M NaOH, where the flow rate was determined empirically by hourly testing on the day of each new inoculation and twice daily for the duration of experimentation.

The chemostat was inoculated with 5 ml of supernatant prepared by resuspending 5 g of human feces in 20 ml of PBS and centrifuging the mixture at 1,800 × *g* for 5 minutes. The chemostat was then run for 15 days to stabilize the composition of the microbial community as previously described (Daisley et al., 2020). To determine how the gut microbiota responded acutely to oxalate, Hungate tubes filled with 7 ml of oxalate maintenance media (1.4 mM K_2_HPO_4_, 1.8 mM KH_2_PO_4_, 3.8 mM (NH_4_)_2_SO_4_, 100 μM MgSO_4•_7H_2_O, 10 mM CH_3_COONa, 4 nM Resazurin, and 0.1% (w/v) yeast extract; Allison et al., 1985), with and without 60 mM sodium oxalate, were inoculated with 500 μl of chemostat contents. Tubes were incubated at 37°C, and at each time point (0, 24, and 48 h), three individual tubes from each group (oxalate or no oxalate) were collected and frozen at-20°C. Microbial DNA was extracted using a PowerSoil-htp 96 Well Soil DNA isolation kit (MoBio, Carlsbad, CA) according to the manufacturer’s protocol, with modifications as outlined by the Earth Microbiome Project.

For 16S rRNA sequencing, amplification of the V4 region of the 16S rRNA gene was carried out as described previously (Al et al., 2018, 2020). Targeted amplification of the 16S rRNA gene V4 region was performed using the established GOLAY-barcoded primers (5′–3′) ACACTCTTTCCCTACACGACGCTCTTCCGATCTNNNNxxxxxxxxxxxxGTGCCAGCMGCCGCGGTAA and (5′–3′) CGGTCTCGGCATTCCTGCTGAACCGCTCTTCCGATCTNNNNxxxxxxxxxxxxGGACTACHVGGGTWTCTAAT, where “xxxxxxxxxxxx” represents the sample-specific 12-mer nucleotide barcode following the Illumina adaptor sequence used for downstream library construction (Caporaso et al., 2012). Sequencing was carried out on the Illumina MiSeq platform at the London Regional Genomics Centre (London, Canada), with the 600 cycle v3 chemistry kit (Illumina, California, USA). Paired-end sequencing was carried out with a 2 × 250 bp cycle profile with 5% PhiX-174 spiked in. The majority of the analyses were carried out in R v4.1.1 (R Core Team, 2021). Reads were trimmed using Cutadapt v3.2 (Martin, 2011), then filtered, merged, and assigned taxonomy using DADA2 v1.20.0 with the SILVA database v138.1 (Quast et al., 2013; Callahan et al., 2016). Amplicon sequence variants (ASVs) were then filtered using a 0.1% maximum cut-off followed by Decontam v1.12.0 (Gloor et al., 2017; Davis et al., 2018). Samples were processed with zCompositions v1.4.0 (Palarea-Albaladejo and Martín-Fernández, 2015) and compared using Adonis2 (R package vegan v2.5–7; Oksanen, et al., 2020). Differential abundance of ASVs was determined using ANCOM v2.1 (Mandal et al., 2015) and confirmed with MaAsLin2 v1.6.0 (Mallick et al., 2021). PICRUSt2 (v2.4.1) was used to infer gene content from taxonomic abundances (Douglas et al., 2020).

### Simulated fecal environment

Strains of *Oxalobacter* (*O. formigenes* HC-1, OxWR1, and HOxSLD-1; *O. aliiformigenes* Ba1, OxK, and HOxNP-2; *Oxalobacter paeniformigenes* OxGP1; and *O. paraformigenes* HOxBLS) were individually cultured together with a fecal inoculum in a colonic medium to determine which strains could persist in the human gut. Colonic medium was prepared as previously described (Liu et al., 2017b) in Hungate tubes. The fecal slurry was prepared by mixing 3 g of fresh feces with 50 ml of pre-reduced PBS + 0.5 g/L L-cysteine in an anaerobic chamber and was filtered through sterile gauze to remove particulate matter. *Oxalobacter* strains were cultured in oxalate broth with 50 mM of oxalate for 2–3 days. Cultures were normalized to 10 Klett units (KS-66 filter; OD_600_ ~ 0.020) using a Klett Photoelectric Colorimeter (Bel-Art Products, catalog number: 37013–0000) with pre-reduced PBS. One milliliter of fecal slurry and 1 ml of normalized *Oxalobacter* culture were inoculated in Hungate tubes containing 11 ml of colonic media. Samples (500–800 μl) were taken every day and frozen at-80°C until DNA extraction.

DNA was extracted using a modified CTAB-based extraction method. Frozen samples were thawed and centrifuged at 18,000 × *g* for 10 min at room temperature. The supernatant was discarded, and the pellet was mixed with 600 μl of CTAB extraction buffer (3.5% hexadecyltrimethylammonium bromide (CTAB), 100 mM tris–HCl [pH 8.0], 10 mM EDTA [pH 8.0], 2.5 M NaCl, and 150 μg/ml proteinase K). The pellet suspension was transferred to bead beating tubes containing 100 μl of 0.1 mm beads and was bead beat for 90 s at 7 m/s. Tubes were transferred to a 56°C water bath for 1 h. Nucleic acid content was purified using one volume of 25:24:1 phenol:chloroform:isoamyl alcohol and washed with one volume of chloroform. Next, one volume of ice-cold isopropanol was added, and the DNA was allowed to precipitate overnight at-20°C. The DNA was washed with 1 ml of 70% ethanol, and the purified DNA was resuspended in nuclease-free water. DNA was diluted 100 × in nuclease-free water before qPCR analysis.

### Design and validation of species-specific qPCR primers

Species-specific qPCR primers were designed using the web-based program RUCS v1.0 (Thomsen et al., 2017). Candidate primers were filtered in R v4.1.2 using a custom script (R Core Team, 2021). Specifically, primers were retained if they had a Tm of 45°C–55°C, two consecutive G or C residues on the 3′ terminator, and three of the last five nucleotides contained either G or C residues. Genus-specific qPCR for *Oxalobacter* (excluding *Oxalobacter vibrioformis* WoOx3; Dehning and Schink, 1989) was based on the consensus sequence of the *oxc* gene using Primer-BLAST (Ye et al., 2012). All primers were tested for potential secondary structures using OligoEvalutator™ (Sigma-Aldrich^8^). Primer-BLAST was used to confirm specificity using the bacteria (taxid: 2) database (Ye et al., 2012). All primers were validated for specificity using approximately 5 ng of genomic DNA from multiple targets and non-target *Oxalobacter* species. Amplification was either undetected or resulted in *C*_T_ values >30 for all valid primer pairs in non-target species. Efficiencies for the *Oxalobacter* genus and species-specific primers were determined to be greater than 90% using the standard curve method. All primer pairs were also run on a 2% agarose gel to confirm amplicon length (Supplementary Figure S1). Supplementary Table S2 lists the final qPCR primer sequences used in this study.

For all qPCR reactions, reagent volumes for a 20 μl reaction (performed in triplicate technical replicates) consisted of 5 μl of DNA, 5 μl of 1.6 μM primer pairs (400 nM final concentration), and 10 μl PowerTrack SYBR Green Master Mix (Thermo Fisher Scientific, catalog number: A46113). Amplification and real-time fluorescence detection were performed on a QuantStudio 5 real-time PCR system (Thermo Fisher Scientific) using the following conditions: 50°C for 2 min, 95°C for 10 min, and 40 cycles of 95°C for 15 s and 60°C for 1 min. Analysis was performed on the associated QuantStudio Design and Analysis software v1.5.2 (Thermo Fisher Scientific). Relative abundance was determined by 2^ΔCt^, where ΔCt was determined by Ct_Calibrator_ – Ct_Target_. The calibrate in all cases was the primer pair 341F/534R of the 16S rRNA gene.

### Data availability statement

Raw sequence reads for whole genome sequencing and 16 s rRNA gene sequencing were uploaded to the NCBI Sequence Read Archive and are accessible under BioProject ID PRJNA836912 and PRJNA841018, respectively. Analysis scripts are available on GitHub (https://github.com/jchmiel4/Oxalobacter_genome_manuscript).

## Results

### Phylogenomic re-evaluation of the *Oxalobacter formigenes* taxon

The genomes of 13 strains named *O. formigenes* and one *O. vibrioformis* strain were sequenced to examine the genetic diversity within the genus. In total, 11 complete assemblies and 3 contiguous assemblies were produced. Quality of the assemblies can be found in Supplementary Table S1. These assemblies were compared with existing *O. formigenes* (GenBank taxid: 2084) genome assemblies for strain characterization. Only genomes with sufficiently high completion and high-quality coverage were kept in the analysis to avoid problems with the absence of certain genes. In total, our analysis included 22 genomes, representing strains isolated from various environments, and included both whole genome and metagenome assemblies (Supplementary Table S1).

**Figure 1:**
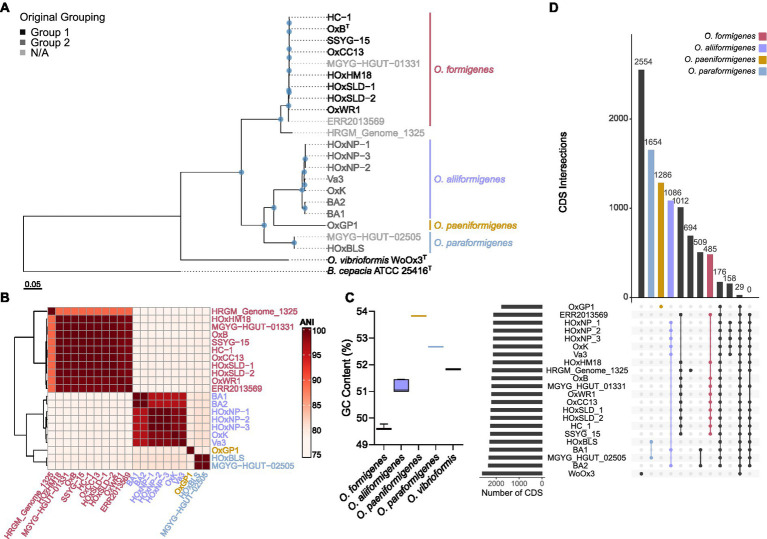
Phylogenetic evaluation of the *Oxalobacter formigenes* taxon. **(A)** Maximum likelihood phylogenetic tree constructed from 691 aligned single-copy core orthologues with *B. cepacia* ATCC 25416 as the outgroup. Greyscale color represents original grouping and colored bars denote new species designations. Scale bar designates mean number of amino acid substitutions per site. Blue circles denote nodes with greater than 80% bootstrap support. **(B)** Heatmap depicting average nucleotide identity pairwise comparisons of strains used in this study. **(C)** Box and whisker plot displaying the whole genome percent GC content of each of the *Oxalobacter* species in this study. Boxes represent first and third quartile values while black line denoting the median, and whiskers encompass maximum and minimum values. **(D)** Coding sequence (CDS) presence/absence plot generated from pangenome. Each column represents the intersection of CDSs in that group (denoted by the number above the column). Filled circles show strains part of the same group. The bars on left show the total number of CDSs present in each genome.

To determine the taxonomy of strains in the genus *Oxalobacter*, 691 single-copy core orthologs were used to generate a high-resolution phylogenetic tree with *B. cepacia* ATCC 25416^T^ as the outgroup (Figure 1A). The tree emphasizes that group I and group II strains are phylogenetically distinct; furthermore, group II strains occur as three divergent clades suggesting that these group II strains represent three novel species of *Oxalobacter* which we have named *Oxalobacter aliiformigenes* sp. nov., *O. paeniformigenes* sp. nov., and *Oxalobacter paraformigenes* sp. nov. (Table 1). The genetic divergence of these new species was confirmed by an average nucleotide identity (ANI) of >95% within each of the four species and an interspecies ANI of 79–81% (Figure 1B). Average amino acid identity (AAI) results corelated with the ANI values and showed an intraspecies similarity of >95% and an interspecies similarity of 67%–84% (Supplementary Figure S2A). Digital DNA–DNA hybridization (dDDH) values for intraspecies ranged from 68% to 100% (with one at ~35%, see discussion below of the *O. formigenes* HRGM_Genome_1325) and for interspecies ranged from 16% to 19% (Supplementary Figure S2B). All raw pairwise comparison values can be found in Supplementary Table S3. GC content was also similar within species (Figure 1C). *Oxalobacter paeniformigenes* has the highest GC content, followed by *O. paraformigenes*, *O. aliiformigenes*, and *O. formigenes*. *Oxalobacter paeniformigenes* was found to have the smallest genome whereas *O. paraformigenes* have the largest (Supplementary Figure S2C).

**Table 1:** New taxonomic designations of *Oxalobacter formigenes* strains.

Strain name	Group	Previous taxonomic designation	New taxonomic designation
OxB^T^	I	*Oxalobacter formigenes*	Unchanged
HC-1	I	*Oxalobacter formigenes*	Unchanged
HOxHM18	I	*Oxalobacter formigenes*	Unchanged
MGYG-HGUT-01331	N/A	*Oxalobacter formigenes*	Unchanged
OxCC13	I	*Oxalobacter formigenes*	Unchanged
OxWR1	I	*Oxalobacter formigenes*	Unchanged
HOxSLD-1	I	*Oxalobacter formigenes*	Unchanged
HOxSLD-2	I	*Oxalobacter formigenes*	Unchanged
SSYG-15	I	*Oxalobacter formigenes*	Unchanged
ERR2013569	N/A	*Oxalobacter formigenes*	Unchanged
HRGM_Genome_1325	N/A	*Oxalobacter formigenes*	Unchanged
BA1	II	*Oxalobacter formigenes*	*Oxalobacter aliiformigenes*
BA2	II	*Oxalobacter formigenes*	*Oxalobacter aliiformigenes*
HOxNP-1	II	*Oxalobacter formigenes*	*Oxalobacter aliiformigenes*
HOxNP-2	II	*Oxalobacter formigenes*	*Oxalobacter aliiformigenes*
HOxNP-3	II	*Oxalobacter formigenes*	*Oxalobacter aliiformigenes*
OxK	II	*Oxalobacter formigenes*	*Oxalobacter aliiformigenes*
Va3	II	*Oxalobacter formigenes*	*Oxalobacter aliiformigenes*
HOxBLS	II	*Oxalobacter formigenes*	*Oxalobacter paraformigenes*
MGYG-HGUT-02505	N/A	*Oxalobacter formigenes*	*Oxalobacter paraformigenes*
OxGP1	II	*Oxalobacter formigenes*	*Oxalobacter paeniformigenes*

N/A, not available.

Computation of the pangenome of all species in the genus *Oxalobacter* indicated that it contains 11,828 genes. This pangenome is comprised of 213 core genes (≥ 95% of strains), 3,717 shell genes (15%–94% of strains), and 7,898 cloud genes (0%–14% of strains). Figure 1D highlights the intersection of CDSs found within species. *O. paraformigenes* shares 1,654 unique genes, while *O. formigenes* strains only share 485 unique genes. No genes were identified that were unique to human-isolated strains, as depicted in the last column of the plot.

**Figure 2:**
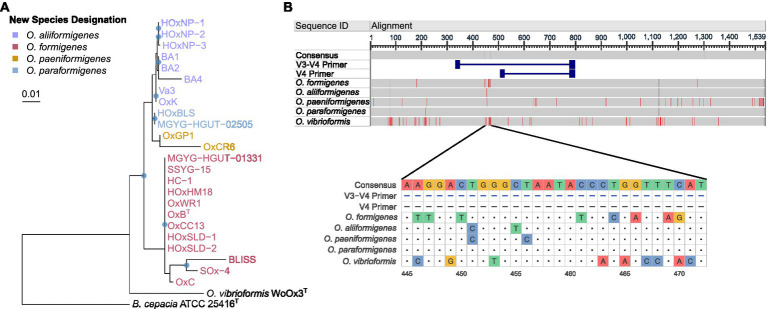
16S rRNA gene phylogenetic analysis of the *Oxalobacter* genus. **(A)** Maximum likelihood phylogram of aligned 16S rRNA gene sequences from *Oxalobacter* with *B. cepacia* ATCC 25416 as the outgroup. Blue circles denote nodes with greater than 50% bootstrap support. Scale bar designates mean number of nucleic acid substitutions per site. **(B)** Alignment of consensus 16S rRNA gene sequences from each species of *Oxalobacter*. Red regions indicate nucleotides different than the than the consensus sequence, which excludes the V3-V4 and V4 primers. Dark gray regions show gaps in the gene. Light gray regions show similarity. Below is a nucleotide level view of the 445–472 bp region of the 16S rRNA gene.

To understand how these taxonomic changes affect strains that do not have whole genome sequences available, we acquired all the 16S rRNA gene sequences from the original groups I and II within *O. formigenes* and constructed a phylogenetic tree with *B. cepacia* ATCC 25416^T^ was used as the outgroup as the outgroup (Figure 2A). The tree highlights that the OxC (accession: U49755), SOx4 (accession: U49751), and BLISS (accession: U49750) strains are likely *O. formigenes*. Strain BA4 (accession: U49749) is placed with *O. aliiformigenes* and OxCR6 (accession: U49754) appears to belong to *O. paeniformigenes*. Alignment of the consensus 16S rRNA gene sequences of each species demonstrated a region of increased diversity at 448–472 bp (Figure 2B). It appears that this region might be best captured using V3-V4 primers for microbiota analysis, but not V4 primers alone.

### Functional annotation of *Oxalobacter* genomes

**Figure 3:**
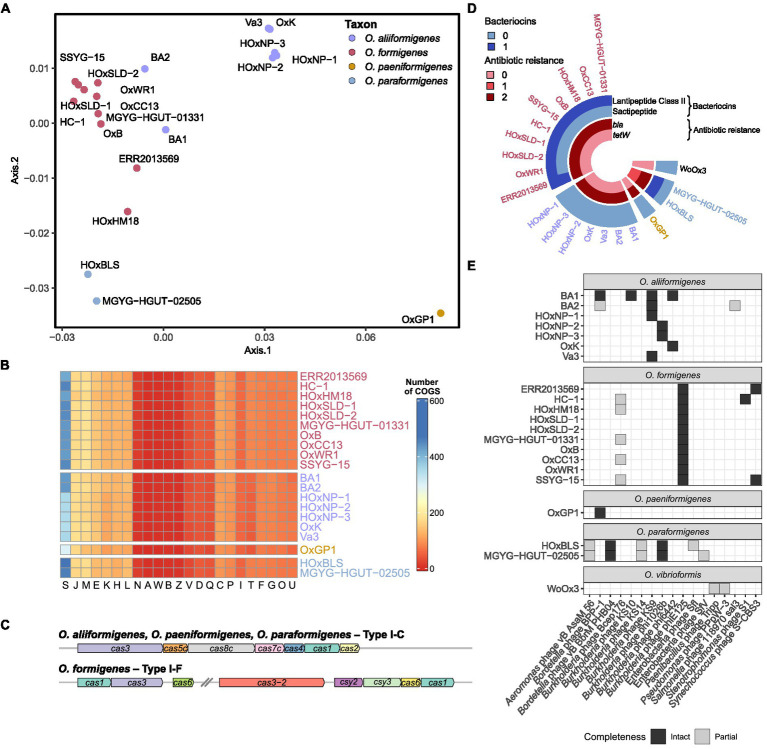
Functional annotation of *Oxalobacter* genomes. **(A)** Principal coordinate analysis (PCoA) plot with Bray–Curtis dissimilarity calculated from absolute abundance of cluster of orthologous group (COG) categories. **(B)** Heatmap displaying that absolute abundance of each COG category. **(C)** Gene arrow maps of Cas clusters found in *Oxalobacter* species. Note *cas8c* gene was not identified in *O. aliiformigenes* OxK and the trailing *cas1* gene was not identified in *O. formigenes* HOxHM18. **(D)** Circular heatmap showing the abundance of bacteriocins and antibiotic resistance genes found in the analyzed *Oxalobacter genomes*. **(E)** Heatmap showing the presence and completeness of prophages found in the analyzed *Oxalobacter* genomes.

Assigning clusters of orthologous groups (COG) categories to the coding sequences for each strain allowed for global functional comparisons between species. Bray–Curtis dissimilarity was calculated from the absolute abundance of COG categories present in each strain and was plotted on a principal coordinate analysis plot for visualization (Figure 3A). Species groupings were found to be statistically different (PERMANOVA; df = 3, r^2^ = 0.81905, *p* = 0.001) and maintained similar homogeneity (PERMDISP; *F* = 1.2586, *p* = 0.3219). Pairwise PERMANOVA revealed clear distinctions between the potential metabolic functions of *O. aliiformigenes* vs. *O. formigenes* and *O. aliiformigenes* vs. *O. paraformigenes* (Supplementary Table S4). Although not significant, there was a sizable distinction between *O. formigenes* vs. *O. paraformigenes*. Visualization of the absolute abundance of each category shows some variability in functional categories but ultimately highlights a notable number of proteins with unknown functions (Group S; Figure 3B).

Next, we examined resistance genes involved in phage resistance, bacteriocin production, and prophages. *O. aliiformigenes*, *O. paeniformigenes*, and *O. paraformigenes* genomes contain a Type I-C Cas cluster, whereas *O. formigenes* encoded for a Type I-F Cas cluster (Figure 3C). A presumed antimicrobial class II lanthipeptide bacteriocin gene was found only in *O. formigenes* strains, and only *O. paraformigenes* strains encoded a sactipeptide (Figure 3D). Antibiotic resistance genes were also queried; all species except for *O. vibrioformis* WoOx3 encode two *bla* genes (β-lactam resistance; Figure 3D). In addition, *O. paraformigenes* strains possess a *tetW* gene for tetracycline resistance. Numerous intact and partial prophages were also identified (Figure 3E). Specifically, all *O. formigenes* strains possess an intact *Burkholderia* phage phiE125, *O. aliiformigenes* strains harbor a diverse repertoire of intact *Burkholderia* prophages (KS9, KS10, phi1026b, and phi6442), the *O. paeniformigenes* OxGP1 genome has a single intact *Bordetella* phage BPP-1, and *O. paraformigenes* strains have an intact *Bordetella* phage vB BbrMPHB04.

**Figure 4:**
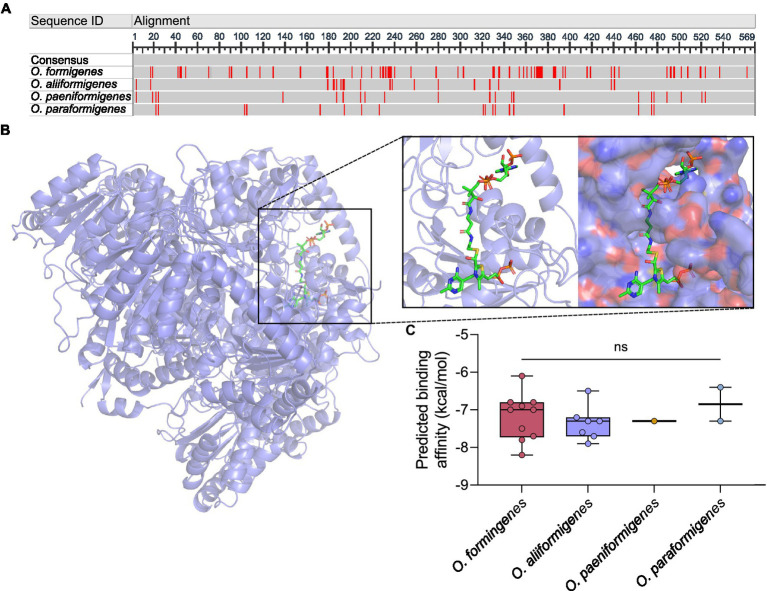
*In silico* analysis of oxalyl-CoA decarboxylase. **(A)** Consensus alignment of the oxalyl-CoA decarboxylase amino acid sequence for each *Oxalobacter* species. Red regions indicate nucleotides different than the consensus sequence. Dark gray regions show gaps in the gene. Light gray regions show similarity. **(B)** Three dimensional *in silico* ribbon model reconstruction of the oxalyl-CoA decarboxylase. Also shown is the binding of the natural ligand, oxalyl-CoA, in a ribbon and space filling structure. **(C)** Predicted binding affinity of each model protein with the natural ligand. Data are displayed as median predicted binding affinity (kcal/mol) and analyzed by one-way ANOVA. In box plot diagrams, circles represent data point, boxes represent first and third quartile values while black lines denote medians, and whiskers encompass maximum and minimum values. ns, not significant.

To gain insight into the functional differences between the species, we investigated the genetic differences in their oxalyl-CoA decarboxylase protein. The alignment of the amino acids revealed some differences in amino acid content (Figure 4A), whereby *O. formigenes* appeared to diverge the most from the consensus sequence. The amino acid sequences of the *oxc* genes were used to create *in silico* model structures of the oxalyl-CoA decarboxylase protein (Figure 4B). The models were subsequently docked with the natural ligand, oxalyl-CoA, and the predicted binding affinity was calculated (Figure 4C). No difference in the predicted binding affinity of oxalyl-CoA was observed between species (Kruskal-Wallis, K-W = 1.521, *p* = 0.7524). Comprehensive model reports for each structure are available in Supplementary Table S5.

### Evaluating the acute microbiota response to oxalate exposure

**Figure 5:**
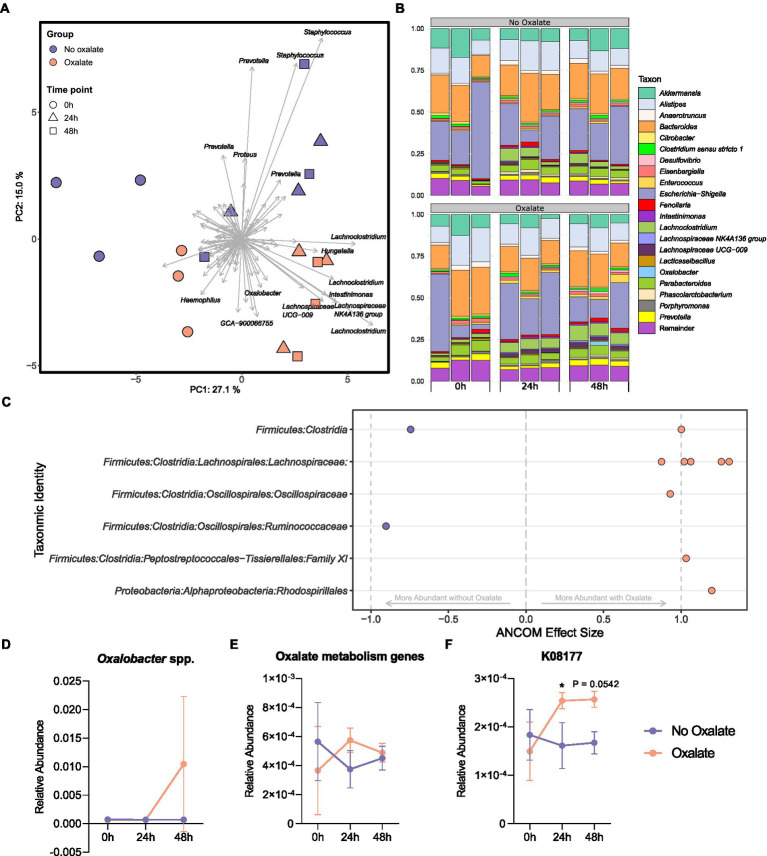
Microbiota analysis of oxalate samples inoculated with chemostat culture. **(A)** Principal component analysis (PCA) plot of ASVs from sample bacterial communities. Centre log ratio-transformed Aitchison distances of sequence variants were used as input values for PCA analysis. Distance between samples on the plot represents differences in microbial community composition. Approximately 42% of the total variance is explained by the first two components shown. Strength and association for sequence variants are depicted by the length and direction of arrows shown. Points are colored by oxalate exposure and shapes denote time point (*n* = 3). **(B)** Relative abundance bar plot of longitudinal samples. Each vertical bar denotes relative sequence variant abundance (collapsed at genus-level identification). Bars are grouped by time point. **(C)** Analysis of differences in sequence variant abundance with oxalate supplementation controlling for the variation associated with time. Positive values indicate sequence variants that were increased with oxalate supplementation and negative values indicate sequence variants that were decreased. Taxa are defined by the most accurate level of classification. Effect size was computed with ANCOM (*W* ≥ 0.7) and supported with MaAsLin2 (Benjamini–Hochberg adjusted *p* < 0.05). **(D)** Longitudinal relative abundance of *Oxalobacter* spp. Data displayed as mean ± SD. Statistical comparisons using two-way ANOVA with Šídák’s multiple comparisons test (*n* = 3). **(E, F)** Relative abundance of predicted oxalate degrading KEGG Orthology numbers over time. Data represents mean ± SD. **(E)** Sum of all predicted oxalate degrading KEGG Orthology numbers (K08177, K01596 [EC:4.1.1.2], K01577 [EC:4.1.1.8], K07749 [EC:2.3.8.16], and K18702 [EC: 2.8.3.19]). **(F)** Abundance of K08177 (major facilitator superfamily transporter, oxalate/formate antiporter family, oxalate/formate antiporter). Statistical comparisons using two-way ANOVA with Šídák’s multiple comparisons test (*n* = 3). *, *p* < 0.05.

To understand how the abundance of *O. formigenes* changes with acute oxalate exposure in a fecal microbial community, a stabilized *ex vivo* chemostat model of the distal gut was inoculated into anaerobic tubes containing oxalate maintenance media with or without 60 mM oxalate for 48 h. The microbial community of oxalate-supplemented samples separated from those without oxalate over time (Figure 5A; PERMANOVA; df = 5, *r*^2^ = 0.497, *p* = 0.002, controlling for time). Figure 5B shows the relative abundance of genera in each sample. Differential abundance testing was used to determine which amplicon sequence variants (ASVs) drove the observed separation between groups (Figure 5C). Controlling for the effects of time, oxalate supplementation increased ASVs in the *Lachnospiraceae*, *Oscillospiraceae*, and Family XI families, as well as the order *Rhodospirillales*. There was a notable decrease in one ASV belonging to the *Ruminococcaceae* family and one to the class *Clostridia*. A slight, albeit not significant, increase in the relative abundance of *Oxalobacter* was also seen over time (Figure 5D; two-way ANOVA, *F* (2, 12) = 2.052, *p* = 0.1712).

Functional inferencing of the 16S rRNA gene sequencing dataset was used to determine how the genetic capacity for oxalate degradation changed over time. The sum of all predicted KEGG Orthology (KO) numbers (K08177, K01596 [EC:4.1.1.2], K01577 [EC:4.1.1.8], K07749 [EC:2.3.8.16], and K18702 [EC: 2.8.3.19]) was not statistically different in oxalate compared with no oxalate groups at each time point (Figure 5E, two-way ANOVA, F (2, 12) = 1.814, *p* = 0.2050). However, changes in the inferred relative abundance of K08177 (oxalate/formate antiporter) was detected (Figure 5F, two-way ANOVA, F (2, 12) = 4.828, *p* = 0.0290). Specifically, after 24 h there was a significant increase in K08177 in the oxalate group (*p* = 0.0457). No other predicted KO numbers associated with oxalate degradation had changed over time (Supplementary Figures S3A–D).

### Species-specific growth in a simulated fecal environment

**Figure 6:**
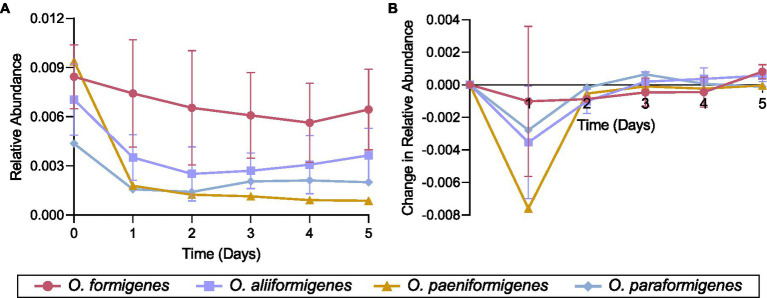
Species-specific growth of *Oxalobacter* in a simulated fecal environment. *Oxalobacter* species were individually inoculated into a simulated fecal environment and subsamples were taken every day for 5 days. **(A)** Relative abundance and **(B)** rate of change in relative abundance of each strain over time as detected by species-specific qPCR analysis. Data are displayed as mean ± SD.

Representative strains for each *Oxalobacter* species were inoculated in a colonic medium with a fecal sample to observe their abundance over time using species-specific qPCR detection. No strains were observed to have a higher relative abundance than the initial inoculum. *Oxalobacter formigenes* strains HC-1, OxWR1, and HOxSLD-1 demonstrated the highest relative abundance throughout the entire time series, while the abundances of other species drastically decreased after the first day of incubation (Figure 6A). *Oxalobacter paeniformigenes* OxGP1 did not have a positive rate of change in abundance at any time point. *O. aliiformigenes* strains Ba1, OxK, and HOxNP-2 demonstrated a positive rate of change in relative abundance from day 3 to day 5, indicating an increase in abundance over these time points (Figure 6B). *O. paraformigenes* HOxBLS had a positive rate of change for days 3 and 4 but it was lost on day 5. *Oxalobacter formigenes* strains HC-1, OxWR1, and HOxSLD-1 did not have a positive rate of change until day 5. As expected, *O. paeniformigenes* OxGP1 did not have a positive rate of change in relative abundance throughout the entire assay. Similar observations were seen using the *oxc* primer pair, which detects *O. formigenes*, *O. aliiformigenes*, *O. paeniformigenes*, and *O. paraformigenes* (Supplementary Figures S4A,B).

## Discussion

This study improves our understanding of the genetic differences between previously acknowledged subgroups of *O. formigenes*. By sequencing the whole genomes of multiple *Oxalobacter* strains, we have identified three novel species within the genus *Oxalobacter*: *O. aliiformigenes* sp. nov., *O. paeniformigenes* sp. nov., and *O. paraformigenes* sp. nov. that are described in more detail below. Functional inferencing demonstrated that these new *Oxalobacter* species present distinct physiological capabilities that have been previously overlooked. Moreover, we found that increased oxalate titres may not be sufficient to stimulate endogenous *Oxalobacter* spp. and promote their abundance. However, exogenous administration of *O. aliiformigenes* could provide a host with strains that can grow alongside the human gut microbiota.

Members of the *O. formigenes* taxon have traditionally fallen into one of the two subgroups based on fatty acid content, 16S rRNA, *oxc* (oxalyl-CoA decarboxylase), and *frc* (formyl-CoA transferase) gene sequence similarities (Allison et al., 1985; Sidhu et al., 1997; Garrity et al., 2005). On the basis of DNA probe identification, Sidhu et al. (1997) suggested that group II strains should be further divided into subgroups. Our work confirms that group II strains are genetically distinct from group I strains, and we also find merit in the subgrouping recognized by Sidhu et al. (1997). However, due to the large differences in ANI, dDDH, and AAI values between these four groups, we have determined that rather than being distinct subgroups of *O. formigenes*, these represent different species within the genus *Oxalobacter*. Differences greater than 1% in the DNA G + C content of these novel species corroborate this observation (Meier-Kolthoff et al., 2014). Uniquely, metagenomic assembly HRGM_Genome_1325 was an outlier in the various distance analyses. This might reflect genome quality; however, the assembly passed our criteria and therefore we still believe that it fits within the *O. formigenes* taxon, but may warrant further consideration as a subspecies within the taxon. While we were not able to sequence the genomes of all *Oxalobacter* strains, the 16S rRNA gene was used to predict the species that additional strains should be assigned to. These results revealed that SOx4, OxC, and BLISS are likely *O. formigenes* strains; BA4 belongs to *O. aliiformigenes*; and OxCR6 is part of *O. paeniformigenes*, which has been similarly shown by others (Sidhu et al., 1997; Daniel et al., 2021). Similarly, the data suggested that strain OxCR6 is related to *O. paeniformigenes*, a relationship that was is supported by the comparable patterns of *oxc* amplification reported previously (Sidhu et al., 1997).

With the identification of novel species, functional differences were determined between groups. Most notable were the differences between *O. formigenes* and the other species. In particular, *O. formigenes* strains contain a Type I-F Cas cluster, while those of the other species contain a Type I-C Cas cluster. *O. formigenes* was also the only species to encode a bacteriocin. Together, these attributes highlight the differences in phage resistance strategies between the species, which could influence their ability to colonize an ecosystem. Given that HOxBLS (*O. paraformigenes*) was commonly used as a representative strain for group II (Daniel et al., 2021), the stark differences between *O. aliiformigenes* and *O. paraformigenes* are also intriguing. Specifically, the significant difference in metabolic and antibiotic resistance genes demonstrates that previous studies making genetic comparisons have likely overlooked the unique functional abilities of *O. aliiformigenes* strains. Another interesting observation was that *O. paeniformigenes* OxGP1 has the smallest number of encoded proteins, implying a potential lack of functional redundancy in its genome. While this work provides some insight into the genes required by *Oxalobacter*, it is complicated by the high proportion (17%–22%) of unannotated genes.

It has been noted that *O. aliiformigenes* is generally more difficult to culture than *O. formigenes* strains (Daniel et al., 2021). Given that the *oxc* gene is critical for metabolism and has been used as a target for genetic identification, we investigated if these genetic differences could alter protein function. Model-based folding and forced ligand docking revealed no change in the predicted binding affinity, suggesting that the fastidious nature of *O. aliiformigenes* comes from other inherent differences that are perhaps related to the diversity or abundance of alternative metabolic genes. While the *frc* gene is also central to oxalate metabolism, it was not modeled due to the genetic redundancy and because the contribution of each gene is unknown. Future work should investigate the differences in substrate utilization and electron acceptor as potential mechanism to explain the slower growth of *O. aliiformigenes*. Notably, the proteome, metabolome, and substrate utilization is well documented in *O. formigenes*, but not in other *Oxalobacter* species (Allison et al., 1985; Ellis et al., 2016; Chamberlain et al., 2019, 2020).

A great deal of effort has been made to bolster *Oxalobacter* colonization through oxalate supplementation or the direct administration of *Oxalobacter* species. Duncan et al., 2002 administered *O. formigenes* HC-1 along with oxalate to promote the strain’s presence in the human gut. We found that directly applying oxalate to a culture of fecal microbes does not increase the abundance of *Oxalobacter* in the first 2 days. This may be because of the fastidious nature of *Oxalobacter*, which is out-competed by more less fastidious microbes that also utilize oxalate; we found that the presence of oxalate resulted in notable increases of *Lachnospiraceae*, as observed previously (Suryavanshi et al., 2016). Though attempts to colonize humans with *Oxalobacter* have been largely unsuccessful (Daniel et al., 2021), we sought to investigate how different *Oxalobacter* species grow alongside commensal gut bacteria. All *Oxalobacter* species drastically decreased in relative abundance during the first 2 days. Remarkably, *O. aliiformigenes* strains began to increase in abundance in the fecal community from day 3 to 5, while *O. formigenes* strains only started to increase from day 5. These data suggest that the introduction of *O. aliiformigenes* strains could lead to their establishment in the gastrointestinal tract. However, it is difficult to ascertain the long-term viability of these strains because the strains were only quantified for 5 days in a closed system. Duncan et al. (2002) found that *O. formigenes* HC-1 had good tolerance toward bile salts but *O. aliiformigenes* Va3 did not. Thus, if *O. aliiformigenes* strains can be delivered in a capsule that releases in the large intestine, they may be able to colonize and flourish. Ultimately, a broader consideration of which *Oxalobacter* species to use as a bacterial intervention to mitigate stone disease is required.

In summary, the findings from this study reveal previously unknown genetic capabilities of members of the genus *Oxalobacter*. The new species identified in this work could be harnessed to develop a bacterial intervention that reduces oxalate and mitigates calcium oxalate stone disease. Attention should be given to *O. aliiformigenes* strains because they can grow alongside other fecal bacteria. However, further studies are needed to explicitly determine the novel metabolic properties of these species and to identify new strains of the underrepresented species.

### Taxonomic and nomenclature proposals

Based on phylogenetic analyses of concatenated single-copy genes, genome comparisons, including average nucleotide identity differences, and genome content comparisons, we propose three new species of *Oxalobacter*. These new taxa were derived from the parent strain *O. formigenes* and were named based on the principles outlined by Pallen et al. (2021); that is, new species retained the parent species name and prefixes were added to denote the taxonomic differences. Physiology is generally the same as described in Allison et al. (1985).

### Description of *Oxalobacter aliiformigenes* sp. nov.

*Oxalobacter aliiformigenes* (a.li.i.for.mi’ge.nes. L. masc. Adj. *alius*, other; N.L. part. Adj. *formigenes*, formic acid producing, and specific epithet of an *Oxalobacter* species; N.L. part. Adj. *aliiformigenes*, meaning that this species is related to but distinct from *O. formigenes*).

Cells are Gram-stain negative, rod-shaped with rounded ends typically measuring 1.6–2.1 × 0.8–1.1 μm on average (estimated from Gram stain), and occurring in singles, pairs, or sometimes in chains. Cells occasionally present as curved. Endospores not found. Flagella not detected. Anaerobic, but aerotolerant with chemotrophic metabolism. Oxalate is used a major carbon and energy source but can be slow growing in oxalate broth. Optimal growth is at 37°C. Indole not formed. Does not appear to reduce nitrate or sulfate. High levels of the fatty acid C19:0 cyclopropane are present and diagnostically useful (Allison et al., 1985). Members of *O. aliiformigenes* can be distinguished from other species in the genus *Oxalobacter* based on phylogenetic analysis and overall genome relatedness indices. Average genome size ranges from 2.2–2.4 Mbp and G + C content of the DNA ranges from 50.9% to 51.5%.

The type strain Va3^T^ (=ATCC XXXX = DSM XXXX) was isolated from a human fecal sample (Duncan et al., 2002).

### Description of *Oxalobacter paeniformigenes* sp. nov.

*Oxalobacter paeniformigenes* (pae.ni.for.mi’ge.nes. L. adv. *Paene*, almost; N.L. part. Adj. *formigenes*, formic acid producing, and specific epithet of an *Oxalobacter* species; N.L. part. Adj. *paeniformigenes*, meaning that this species is related to but distinct from *O. formigenes*).

Cells are Gram-stain negative, rod-shaped with rounded ends typically measuring 1.4–2.2 × 0.8–1.2 μm on average (estimated from Gram stain), and occurring in singles, pairs, or sometimes in chains. Cells occasionally present as curved. Endospores not found. Flagella not detected. Anaerobic, but aerotolerant with chemotrophic metabolism. Oxalate is used a major carbon and energy source. Optimal growth is at 37°C. Indole not formed. Does not appear to reduce nitrate or sulfate. Members of *O. paeniformigenes* can be distinguished from other species in the genus *Oxalobacter* based on phylogenetic analysis and overall genome relatedness indices. The genome size of the type strain is 1.93 Mb and the G + C content is 53.8%.

The type strain OxGP1^T^ (=ATCC XXXX = DSM XXXX) was isolated from guinea pig cecum.

### Description of *Oxalobacter paraformigenes* sp. nov.

*Oxalobacter paraformigenes* (pa.ra.for.mi’ge.nes. Gr. pref. *Para*, beside; N.L. part. Adj. *formigenes*, formic acid producing, and specific epithet of an *Oxalobacter* species; N.L. part. Adj. *paraformigenes*, meaning that this species is related to but distinct from *Oxalobacter formigenes*).

Cells are Gram-stain negative, rod-shaped with rounded ends typically measuring 1.6–2.4 × 0.8–1.1 μm on average (estimated from Gram stain), and occurring in singles, pairs, or sometimes in chains. Cells occasionally present as curved. Endospores not found. Flagella not detected. Anaerobic, but aerotolerant with chemotrophic metabolism. Oxalate is used a major carbon and energy source. Optimal growth is at 37°C. Indole not formed. Does not appear to reduce nitrate or sulfate. Members of *O. paraformigenes* can be distinguished from other species in the *Oxalobacter* genus based on phylogenetic analysis and overall genome relatedness indices. The genome size of the type strain is 2.49 Mb and the G + C content is 52.7%.

The type strain *O. paraformigenes* HOxBLS^T^ (=ATCC XXXX = DSM XXXX) was isolated from human fecal material.

## Data availability statement

The datasets presented in this study can be found in online repositories. The names of the repository/repositories and accession number(s) can be found at: https://www.ncbi.nlm.nih.gov/, PRJNA836912; https://www.ncbi.nlm.nih.gov/, PRJNA841018.

## Ethics statement

The studies involving human participants were reviewed and approved by Health Sciences Research Ethics Board at the University of Western Ontario. The patients/participants provided their written informed consent to participate in this study.

## Author contributions

JC, RV, RC, and JPB conceived the study. JC, GS, RV, RC, DG, HS, and AK carried out the physical experiments. JC, CC, GS, DG, KA, SADR, and PA carried out the computational analysis. JC, CC, GS, and KA wrote the original manuscript and designed the figures. JC, SK, and JPB contributed to the novel species designations. VT and SK provided significant taxonomic consultation and judgment. HR and JB provided clinical feedback and applicability. All authors contributed to the article and approved the submitted version.

## Funding

This work is supported by a Lawson Health Research Institute Internal Research Fund, a Northeastern Section of the American Urological Association Datta G. Wagle Young Investigator Grant, Weston Family Foundation, and a Canadian Institutes of Health Research Canada Graduate Scholarship–Doctoral (175836).

## Conflict of interest

The authors declare that the research was conducted in the absence of any commercial or financial relationships that could be construed as a potential conflict of interest.

## Publisher’s note

All claims expressed in this article are solely those of the authors and do not necessarily represent those of their affiliated organizations, or those of the publisher, the editors and the reviewers. Any product that may be evaluated in this article, or claim that may be made by its manufacturer, is not guaranteed or endorsed by the publisher.

## Footnotes

^1^ https://ggdc.dsmz.de/ggdc.php#

^2^ http://eggnog-mapper.embl.de/

^3^ https://crisprcas.i2bc.paris-saclay.fr/CrisprCasFinder/Index

^4^ http://bagel4.molgenrug.nl/

^5^ https://phaster.ca/

^6^ https://www.ebi.ac.uk/Tools/msa/emboss_cons/

^7^ https://www.ncbi.nlm.nih.gov/projects/msaviewer/

^8^ http://www.oligoevaluator.com/LoginServlet

## References

[ref1] Al K. F. Bisanz J. E. Gloor G. B. Reid G. Burton J. P. (2018). Evaluation of sampling and storage procedures on preserving the community structure of stool microbiota: a simple at-home toilet-paper collection method. J. Microbiol. Methods 144, 117–121. doi: 10.1016/j.mimet.2017.11.014, PMID: 29155236

[ref2] Al K. F. Denstedt J. D. Daisley B. A. Bjazevic J. Welk B. K. Pautler S. E.. (2020). Ureteral stent microbiota is associated with patient comorbidities but not antibiotic exposure. Cell Rep. Med. 1:100094. doi: 10.1016/j.xcrm.2020.100094, PMID: 33205072 PMC7659606

[ref3] Allison M. J. Dawson K. A. Mayberry W. R. Foss J. G. (1985). *Oxalobacter formigenes* gen. Nov., sp. nov.: oxalate-degrading anaerobes that inhabit the gastrointestinal tract. Arch. Microbiol. 141, 1–7. doi: 10.1007/BF00446731, PMID: 3994481

[ref4] Andrews S. (2010). FastQC: a quality control tool for high throughput sequence data. Available at: https://www.bioinformatics.babraham.ac.uk/projects/fastqc/ (Accessed June 11, 2022).

[ref5] Arndt D. Grant J. R. Marcu A. Sajed T. Pon A. Liang Y.. (2016). PHASTER: a better, faster version of the PHAST phage search tool. Nucleic Acids Res. 44, W16–W21. doi: 10.1093/nar/gkw387, PMID: 27141966 PMC4987931

[ref6] Baker P. R. S. Cramer S. D. Kennedy M. Assimos D. G. Holmes R. P. (2004). Glycolate and glyoxylate metabolism in HepG2 cells. Am. J. Physiol. Cell Physiol. 287, C1359–C1365. doi: 10.1152/ajpcell.00238.2004, PMID: 15240345

[ref7] Bankevich A. Nurk S. Antipov D. Gurevich A. A. Dvorkin M. Kulikov A. S.. (2012). SPAdes: a new genome assembly algorithm and its applications to single-cell sequencing. J. Comput. Biol. 19, 455–477. doi: 10.1089/cmb.2012.0021, PMID: 22506599 PMC3342519

[ref8] Barnett C. Nazzal L. Goldfarb D. S. Blaser M. J. (2016). The presence of *Oxalobacter formigenes* in the microbiome of healthy young adults. J. Urol. 195, 499–506. doi: 10.1016/j.juro.2015.08.070, PMID: 26292041 PMC4747808

[ref9] Benkert P. Biasini M. Schwede T. (2011). Toward the estimation of the absolute quality of individual protein structure models. Bioinformatics 27, 343–350. doi: 10.1093/bioinformatics/btq662, PMID: 21134891 PMC3031035

[ref10] Berthold C. L. Toyota C. G. Moussatche P. Wood M. D. Leeper F. Richards N. G. J.. (2007). Crystallographic snapshots of oxalyl-CoA decarboxylase give insights into catalysis by nonoxidative ThDP-dependent decarboxylases. Structure 15, 853–861. doi: 10.1016/j.str.2007.06.001, PMID: 17637344

[ref11] Bertoni M. Kiefer F. Biasini M. Bordoli L. Schwede T. (2017). Modeling protein quaternary structure of homo-and hetero-oligomers beyond binary interactions by homology. Sci. Rep. 7:10480. doi: 10.1038/s41598-017-09654-8 28874689 PMC5585393

[ref12] Biasini M. Bienert S. Waterhouse A. Arnold K. Studer G. Schmidt T.. (2014). SWISS-MODEL: modelling protein tertiary and quaternary structure using evolutionary information. Nucleic Acids Res. 42, W252–W258. doi: 10.1093/nar/gku340, PMID: 24782522 PMC4086089

[ref13] Callahan B. J. McMurdie P. J. Rosen M. J. Han A. W. Johnson A. J. A. Holmes S. P. (2016). DADA2: high-resolution sample inference from Illumina amplicon data. Nat. Methods 13, 581–583. doi: 10.1038/nmeth.3869, PMID: 27214047 PMC4927377

[ref14] Camacho C. Coulouris G. Avagyan V. Ma N. Papadopoulos J. Bealer K.. (2009). BLAST+: architecture and applications. BMC Bioinformatics 10:421. doi: 10.1186/1471-2105-10-421 20003500 PMC2803857

[ref15] Capella-Gutiérrez S. Silla-Martínez J. M. Gabaldón T. (2009). trimAl: a tool for automated alignment trimming in large-scale phylogenetic analyses. Bioinformatics 25, 1972–1973. doi: 10.1093/bioinformatics/btp348, PMID: 19505945 PMC2712344

[ref16] Caporaso J. G. Lauber C. L. Walters W. A. Berg-Lyons D. Huntley J. Fierer N.. (2012). Ultra-high-throughput microbial community analysis on the Illumina HiSeq and MiSeq platforms. ISME J. 6, 1621–1624. doi: 10.1038/ismej.2012.8, PMID: 22402401 PMC3400413

[ref17] Chamberlain C. A. Hatch M. Garrett T. J. (2019). Metabolomic and lipidomic characterization of *Oxalobacter formigenes* strains HC1 and OxWR by UHPLC-HRMS. Anal. Bioanal. Chem. 411, 4807–4818. doi: 10.1007/s00216-019-01639-y, PMID: 30740635 PMC6612311

[ref18] Chamberlain C. A. Hatch M. Garrett T. J. (2020). *Oxalobacter formigenes* produces metabolites and lipids undetectable in oxalotrophic *Bifidobacterium animalis*. Metabolomics 16:122. doi: 10.1007/s11306-020-01747-2 33219444

[ref19] Chen S. Zhou Y. Chen Y. Gu J. (2018). Fastp: an ultra-fast all-in-one FASTQ preprocessor. Bioinformatics 34, i884–i890. doi: 10.1093/bioinformatics/bty560, PMID: 30423086 PMC6129281

[ref20] Conway J. R. Lex A. Gehlenborg N. (2017). UpSetR: an R package for the visualization of intersecting sets and their properties. Bioinformatics 33, 2938–2940. doi: 10.1093/bioinformatics/btx364, PMID: 28645171 PMC5870712

[ref21] Cornick N. A. Allison M. J. (1996). Assimilation of oxalate, acetate, and CO_2_ by *Oxalobacter formigenes*. Can. J. Microbiol. 42, 1081–1086. doi: 10.1139/m96-138, PMID: 8941983

[ref22] Couvin D. Bernheim A. Toffano-Nioche C. Touchon M. Michalik J. Néron B.. (2018). CRISPRCasFinder, an update of CRISRFinder, includes a portable version, enhanced performance and integrates search for Cas proteins. Nucleic Acids Res. 46, W246–W251. doi: 10.1093/nar/gky425, PMID: 29790974 PMC6030898

[ref23] Daisley B. A. Chanyi R. M. Abdur-Rashid K. Al K. F. Gibbons S. Chmiel J. A.. (2020). Abiraterone acetate preferentially enriches for the gut commensal *Akkermansia muciniphila* in castrate-resistant prostate cancer patients. Nat. Commun. 11:4822. doi: 10.1038/s41467-020-18649-5 32973149 PMC7515896

[ref24] Daniel S. L. Moradi L. Paiste H. Wood K. D. Assimos D. G. Holmes R. P.. (2021). Forty years of *Oxalobacter formigenes*, a gutsy oxalate-degrading specialist. Appl. Environ. Microbiol. 87:e0054421. doi: 10.1128/AEM.00544-21, PMID: 34190610 PMC8388816

[ref25] Davis N. M. Proctor D. M. Holmes S. P. Relman D. A. Callahan B. J. (2018). Simple statistical identification and removal of contaminant sequences in marker-gene and metagenomics data. Microbiome 6:226. doi: 10.1186/s40168-018-0605-2 30558668 PMC6298009

[ref26] Dawson K. A. Allison M. J. Hartman P. A. (1980). Isolation and some characteristics of anaerobic oxalate-degrading bacteria from the rumen. Appl. Environ. Microbiol. 40, 833–839. doi: 10.1128/aem.40.4.833-839.1980, PMID: 7425628 PMC291667

[ref27] De Coster W. D’Hert S. Schultz D. T. Cruts M. Van Broeckhoven C. (2018). NanoPack: visualizing and processing long-read sequencing data. Bioinformatics 34, 2666–2669. doi: 10.1093/bioinformatics/bty149, PMID: 29547981 PMC6061794

[ref28] Dehning I. Schink B. (1989). Two new species of anaerobic oxalate-fermenting bacteria, *Oxalobacter vibrioformis* sp. nov. and *clostridium oxalicum* sp. nov., from sediment samples. Arch. Microbiol. 153, 79–84. doi: 10.1007/BF00277545

[ref29] Douglas G. M. Maffei V. J. Zaneveld J. R. Yurgel S. N. Brown J. R. Taylor C. M.. (2020). PICRUSt2 for prediction of metagenome functions. Nat. Biotechnol. 38, 685–688. doi: 10.1038/s41587-020-0548-6, PMID: 32483366 PMC7365738

[ref30] Duncan S. H. Richardson A. J. Kaul P. Holmes R. P. Allison M. J. Stewart C. S. (2002). *Oxalobacter formigenes* and its potential role in human health. Appl. Environ. Microbiol. 68, 3841–3847. doi: 10.1128/AEM.68.8.3841-3847.2002, PMID: 12147479 PMC124017

[ref31] Edgar R. C. (2021). MUSCLE v5 enables improved estimates of phylogenetic tree confidence by ens^1^emble bootstrapping [Preprint]. doi: 10.1101/2021.06.20.449169

[ref32] Ellis M. E. Mobley J. A. Holmes R. P. Knight J. (2016). Proteome dynamics of the specialist oxalate degrader *Oxalobacter formigenes*. J. Proteomics Bioinform. 9, 19–24. doi: 10.4172/jpb.1000384, PMID: 26924912 PMC4764995

[ref33] Emms D. M. Kelly S. (2019). OrthoFinder: phylogenetic orthology inference for comparative genomics. Genome Biol. 20:238. doi: 10.1186/s13059-019-1832-y 31727128 PMC6857279

[ref34] Feldgarden M. Brover V. Gonzalez-Escalona N. Frye J. G. Haendiges J. Haft D. H.. (2021). AMRFinderPlus and the reference gene Catalog facilitate examination of the genomic links among antimicrobial resistance, stress response, and virulence. Sci. Rep. 11:12728. doi: 10.1038/s41598-021-91456-0 34135355 PMC8208984

[ref35] Garrity G. M. Bell J. A. Lilburn T. (2005). “Class II. Betaproteobacteria class. Nov” in Bergey’s Manual® of Systematic Bacteriology: Volume Two the Proteobacteria Part C the Alpha-, Beta-, Delta-, and Epsilonproteobacteria. eds. Brenner D. J. Krieg N. R. Staley J. T. (Boston, MA: Springer), 575–922.

[ref36] Gloor G. B. Macklaim J. M. Pawlowsky-Glahn V. Egozcue J. J. (2017). Microbiome datasets are compositional: and this is not optional. Front. Microbiol. 8, 1–6. doi: 10.3389/fmicb.2017.02224 29187837 PMC5695134

[ref37] Gu Z. Eils R. Schlesner M. (2016). Complex heatmaps reveal patterns and correlations in multidimensional genomic data. Bioinformatics 32, 2847–2849. doi: 10.1093/bioinformatics/btw313, PMID: 27207943

[ref38] Gu Z. Gu L. Eils R. Schlesner M. Brors B. (2014). Circlize implements and enhances circular visualization in R. Bioinformatics 30, 2811–2812. doi: 10.1093/bioinformatics/btu393, PMID: 24930139

[ref39] Gurevich A. Saveliev V. Vyahhi N. Tesler G. (2013). QUAST: quality assessment tool for genome assemblies. Bioinformatics 29, 1072–1075. doi: 10.1093/bioinformatics/btt086, PMID: 23422339 PMC3624806

[ref40] Hoppe B. Beck B. Gatter N. von Unruh G. Tischer A. Hesse A.. (2006). *Oxalobacter formigenes*: a potential tool for the treatment of primary hyperoxaluria type 1. Kidney Int. 70, 1305–1311. doi: 10.1038/sj.ki.5001707, PMID: 16850020

[ref41] Hoppe B. Niaudet P. Salomon R. Harambat J. Hulton S.-A. Van’t Hoff W.. (2017). A randomised phase I/II trial to evaluate the efficacy and safety of orally administered *Oxalobacter formigenes* to treat primary hyperoxaluria. Pediatr. Nephrol. 32, 781–790. doi: 10.1007/s00467-016-3553-8, PMID: 27924398

[ref42] Huerta-Cepas J. Forslund K. Coelho L. P. Szklarczyk D. Jensen L. J. von Mering C.. (2017). Fast genome-wide functional annotation through orthology assignment by eggNOG-mapper. Mol. Biol. Evol. 34, 2115–2122. doi: 10.1093/molbev/msx148, PMID: 28460117 PMC5850834

[ref43] Huerta-Cepas J. Szklarczyk D. Heller D. Hernández-Plaza A. Forslund S. K. Cook H.. (2019). eggNOG 5.0: a hierarchical, functionally and phylogenetically annotated orthology resource based on 5090 organisms and 2502 viruses. Nucleic Acids Res. 47, D309–D314. doi: 10.1093/nar/gky1085, PMID: 30418610 PMC6324079

[ref44] Hungate R. E. (1950). The anaerobic mesophiliccellulolytic bacteria. Bacteriol. Rev. 14, 1–49. doi: 10.1128/br.14.1.1-49.1950, PMID: 15420122 PMC440953

[ref45] Hyatt D. Chen G.-L. Locascio P. F. Land M. L. Larimer F. W. Hauser L. J. (2010). Prodigal: prokaryotic gene recognition and translation initiation site identification. BMC Bioinformatics 11:119. doi: 10.1186/1471-2105-11-119 20211023 PMC2848648

[ref46] Jain C. Rodriguez-R L. M. Phillippy A. M. Konstantinidis K. T. Aluru S. (2018). High throughput ANI analysis of 90K prokaryotic genomes reveals clear species boundaries. Nat. Commun. 9:5114. doi: 10.1038/s41467-018-07641-9 30504855 PMC6269478

[ref47] Katoh K. Standley D. M. (2013). MAFFT multiple sequence alignment software version 7: improvements in performance and usability. Mol. Biol. Evol. 30, 772–780. doi: 10.1093/molbev/mst010, PMID: 23329690 PMC3603318

[ref48] Kaufman D. W. Kelly J. P. Curhan G. C. Anderson T. E. Dretler S. P. Preminger G. M.. (2008). *Oxalobacter formigenes* may reduce the risk of calcium oxalate kidney stones. J. Am. Soc. Nephrol. 19, 1197–1203. doi: 10.1681/ASN.2007101058, PMID: 18322162 PMC2396938

[ref49] Kolde R. (2019). Pheatmap: Pretty Heatmaps. Available at: https://CRAN.R-project.org/package=pheatmap (Accessed February 22, 2022).

[ref50] Kolmogorov M. Yuan J. Lin Y. Pevzner P. A. (2019). Assembly of long, error-prone reads using repeat graphs. Nat. Biotechnol. 37, 540–546. doi: 10.1038/s41587-019-0072-8, PMID: 30936562

[ref51] Lange J. N. Wood K. D. Wong H. Otto R. Mufarrij P. W. Knight J.. (2012). Sensitivity of human strains of *Oxalobacter formigenes* to commonly prescribed antibiotics. Urology 79, 1286–1289. doi: 10.1016/j.urology.2011.11.017, PMID: 22656407 PMC3569510

[ref52] Li H. (2018). Minimap2: pairwise alignment for nucleotide sequences. Bioinformatics 34, 3094–3100. doi: 10.1093/bioinformatics/bty191, PMID: 29750242 PMC6137996

[ref53] Liu M. Devlin J. C. Hu J. Volkova A. Battaglia T. W. Ho M.. (2021). Microbial genetic and transcriptional contributions to oxalate degradation by the gut microbiota in health and disease. Elife 10:e63642. doi: 10.7554/eLife.63642, PMID: 33769280 PMC8062136

[ref54] Liu M. Koh H. Kurtz Z. D. Battaglia T. PeBenito A. Li H.. (2017a). *Oxalobacter formigenes*-associated host features and microbial community structures examined using the American gut project. Microbiome 5:108. doi: 10.1186/s40168-017-0316-0 28841836 PMC5571629

[ref55] Liu Y. Gibson G. R. Walton G. E. (2017b). A three-stage continuous culture approach to study the impact of probiotics, prebiotics and fat intake on faecal microbiota relevant to an over 60s population. J. Funct. Foods 32, 238–247. doi: 10.1016/j.jff.2017.02.035

[ref56] Mallick H. Rahnavard A. McIver L. J. Ma S. Zhang Y. Nguyen L. H.. (2021). Multivariable association discovery in population-scale meta-omics studies. PLoS Comput. Biol. 17:e1009442. doi: 10.1371/journal.pcbi.1009442, PMID: 34784344 PMC8714082

[ref57] Mandal S. Van Treuren W. White R. A. Eggesbø M. Knight R. Peddada S. D. (2015). Analysis of composition of microbiomes: a novel method for studying microbial composition. Microb. Ecol. Health Dis. 26:27663. doi: 10.3402/mehd.v26.27663, PMID: 26028277 PMC4450248

[ref58] Martin M. (2011). Cutadapt removes adapter sequences from high-throughput sequencing reads. EMBne J. 17, 10–12. doi: 10.14806/ej.17.1.200

[ref59] Martinez Arbizu P. (2020). pairwiseAdonis: Pairwise Multilevel Comparison Using Adonis. Available at: https://github.com/pmartinezarbizu/pairwiseAdonis (Accessed May 30, 2022).

[ref60] Massey L. K. Roman-Smith H. Sutton R. A. (1993). Effect of dietary oxalate and calcium on urinary oxalate and risk of formation of calcium oxalate kidney stones. J. Am. Diet. Assoc. 93, 901–906. doi: 10.1016/0002-8223(93)91530-4 8335871

[ref61] McDonald J. A. K. Schroeter K. Fuentes S. Heikamp-Dejong I. Khursigara C. M. de Vos W. M.. (2013). Evaluation of microbial community reproducibility, stability and composition in a human distal gut chemostat model. J. Microbiol. Methods 95, 167–174. doi: 10.1016/j.mimet.2013.08.008, PMID: 23994646

[ref62] Meier-Kolthoff J. P. Carbasse J. S. Peinado-Olarte R. L. Göker M. (2022). TYGS and LPSN: a database tandem for fast and reliable genome-based classification and nomenclature of prokaryotes. Nucleic Acids Res. 50, D801–D807. doi: 10.1093/nar/gkab902, PMID: 34634793 PMC8728197

[ref63] Meier-Kolthoff J. P. Klenk H.-P. Göker M. (2014). Taxonomic use of DNA G+C content and DNA-DNA hybridization in the genomic age. Int. J. Syst. Evol. Microbiol. 64, 352–356. doi: 10.1099/ijs.0.056994-0, PMID: 24505073

[ref64] Milliner D. Hoppe B. Groothoff J. (2018). A randomised phase II/III study to evaluate the efficacy and safety of orally administered *Oxalobacter formigenes* to treat primary hyperoxaluria. Urolithiasis 46, 313–323. doi: 10.1007/s00240-017-0998-6, PMID: 28718073 PMC6061479

[ref65] Oksanen J. Blanchet G. Friendly M. Kindt R. Legendre P. McGlinn D.. (2020). Vegan: community ecology package. Available at: https://CRAN.R-project.org/package=vegan (Accessed December 16, 2021).

[ref66] Page A. J. Cummins C. A. Hunt M. Wong V. K. Reuter S. Holden M. T. G.. (2015). Roary: rapid large-scale prokaryote pan genome analysis. Bioinformatics 31, 3691–3693. doi: 10.1093/bioinformatics/btv421, PMID: 26198102 PMC4817141

[ref67] Palarea-Albaladejo J. Martín-Fernández J. A. (2015). zCompositions — R package for multivariate imputation of left-censored data under a compositional approach. Chemom. Intell. Lab. Syst. 143, 85–96. doi: 10.1016/j.chemolab.2015.02.019

[ref68] Pallen M. J. Telatin A. Oren A. (2021). The next million names for Archaea and bacteria. Trends Microbiol. 29, 289–298. doi: 10.1016/j.tim.2020.10.009, PMID: 33288384

[ref69] Parks D. H. Imelfort M. Skennerton C. T. Hugenholtz P. Tyson G. W. (2015). CheckM: assessing the quality of microbial genomes recovered from isolates, single cells, and metagenomes. Genome Res. 25, 1043–1055. doi: 10.1101/gr.186072.114, PMID: 25977477 PMC4484387

[ref70] Quast C. Pruesse E. Yilmaz P. Gerken J. Schweer T. Yarza P.. (2013). The SILVA ribosomal RNA gene database project: improved data processing and web-based tools. Nucleic Acids Res. 41, D590–D596. doi: 10.1093/nar/gks1219, PMID: 23193283 PMC3531112

[ref71] R Core Team (2021). R: A language and environment for statistical computing. Vienna, Austria: R Foundation for Statistical Computing. Available at: https://www.R-project.org/ (Accessed November 6, 2022).

[ref72] Romero V. Akpinar H. Assimos D. G. (2010). Kidney stones: a global picture of prevalence, incidence, and associated risk factors. Rev. Urol. 12, e86–e96. 20811557 PMC2931286

[ref73] Scales C. D. Smith A. C. Hanley J. M. Saigal C. S. Urologic Diseases in America Project (2012). Prevalence of kidney stones in the United States. Eur. Urol. 62, 160–165. doi: 10.1016/j.eururo.2012.03.052, PMID: 22498635 PMC3362665

[ref74] Seemann T. (2014). Prokka: rapid prokaryotic genome annotation. Bioinformatics 30, 2068–2069. doi: 10.1093/bioinformatics/btu153, PMID: 24642063

[ref75] Sidhu H. Allison M. Peck A. B. (1997). Identification and classification of *Oxalobacter formigenes* strains by using oligonucleotide probes and primers. J. Clin. Microbiol. 35, 350–353. doi: 10.1128/jcm.35.2.350-353.1997, PMID: 9003594 PMC229578

[ref76] Singh P. Enders F. T. Vaughan L. E. Bergstralh E. J. Knoedler J. J. Krambeck A. E.. (2015). Stone composition among first-time symptomatic kidney stone formers in the community. Mayo Clin. Proc. 90, 1356–1365. doi: 10.1016/j.mayocp.2015.07.016, PMID: 26349951 PMC4593754

[ref77] Smith R. L. Strohmaier F. E. Oremland R. S. (1985). Isolation of anaerobic oxalate-degrading bacteria from freshwater lake sediments. Arch. Microbiol. 141, 8–13. doi: 10.1007/BF00446732

[ref78] Stamatakis A. (2014). RAxML version 8: a tool for phylogenetic analysis and post-analysis of large phylogenies. Bioinformatics 30, 1312–1313. doi: 10.1093/bioinformatics/btu033, PMID: 24451623 PMC3998144

[ref79] Steinegger M. Meier M. Mirdita M. Vöhringer H. Haunsberger S. J. Söding J. (2019). HH-suite3 for fast remote homology detection and deep protein annotation. BMC Bioinformatics 20:473. doi: 10.1186/s12859-019-3019-7 31521110 PMC6744700

[ref80] Studer G. Rempfer C. Waterhouse A. M. Gumienny R. Haas J. Schwede T. (2020). QMEANDisCo-distance constraints applied on model quality estimation. Bioinformatics 36, 1765–1771. doi: 10.1093/bioinformatics/btz828, PMID: 31697312 PMC7075525

[ref81] Suryavanshi M. V. Bhute S. S. Jadhav S. D. Bhatia M. S. Gune R. P. Shouche Y. S. (2016). Hyperoxaluria leads to dysbiosis and drives selective enrichment of oxalate metabolizing bacterial species in recurrent kidney stone endures. Sci. Rep. 6:34712. doi: 10.1038/srep34712 27708409 PMC5052600

[ref82] Tang R. Jiang Y. Tan A. Ye J. Xian X. Xie Y.. (2018). 16S rRNA gene sequencing reveals altered composition of gut microbiota in individuals with kidney stones. Urolithiasis 46, 503–514. doi: 10.1007/s00240-018-1037-y, PMID: 29353409

[ref83] Thomsen M. C. F. Hasman H. Westh H. Kaya H. Lund O. (2017). RUCS: rapid identification of PCR primers for unique core sequences. Bioinformatics 33, 3917–3921. doi: 10.1093/bioinformatics/btx526, PMID: 28968748 PMC5860091

[ref84] Ticinesi A. Milani C. Guerra A. Allegri F. Lauretani F. Nouvenne A.. (2018). Understanding the gut-kidney axis in nephrolithiasis: an analysis of the gut microbiota composition and functionality of stone formers. Gut 67, 2097–2106. doi: 10.1136/gutjnl-2017-315734, PMID: 29705728

[ref85] Trott O. Olson A. J. (2010). AutoDock Vina: improving the speed and accuracy of docking with a new scoring function, efficient optimization, and multithreading. J. Comput. Chem. 31, 455–461. doi: 10.1002/jcc.21334, PMID: 19499576 PMC3041641

[ref86] Turney B. W. Reynard J. M. Noble J. G. Keoghane S. R. (2012). Trends in urological stone disease. BJU Int. 109, 1082–1087. doi: 10.1111/j.1464-410X.2011.10495.x, PMID: 21883851

[ref87] van Heel A. J. de Jong A. Song C. Viel J. H. Kok J. Kuipers O. P. (2018). BAGEL4: a user-friendly web server to thoroughly mine RiPPs and bacteriocins. Nucleic Acids Res. 46, W278–W281. doi: 10.1093/nar/gky383, PMID: 29788290 PMC6030817

[ref88] Vaser R. Sović I. Nagarajan N. Šikić M. (2017). Fast and accurate *de novo* genome assembly from long uncorrected reads. Genome Res. 27, 737–746. doi: 10.1101/gr.214270.116, PMID: 28100585 PMC5411768

[ref89] Wang W. Fan J. Huang G. Li J. Zhu X. Tian Y.. (2017). Prevalence of kidney stones in mainland China: a systematic review. Sci. Rep. 7:41630. doi: 10.1038/srep41630 28139722 PMC5282506

[ref90] Waterhouse A. Bertoni M. Bienert S. Studer G. Tauriello G. Gumienny R.. (2018). SWISS-MODEL: homology modelling of protein structures and complexes. Nucleic Acids Res. 46, W296–W303. doi: 10.1093/nar/gky427, PMID: 29788355 PMC6030848

[ref91] Whittamore J. M. Hatch M. (2017). The role of intestinal oxalate transport in hyperoxaluria and the formation of kidney stones in animals and man. Urolithiasis 45, 89–108. doi: 10.1007/s00240-016-0952-z, PMID: 27913853 PMC5358548

[ref92] Wick R. R. Judd L. M. Gorrie C. L. Holt K. E. (2017). Unicycler: resolving bacterial genome assemblies from short and long sequencing reads. PLoS Comput. Biol. 13:e1005595. doi: 10.1371/journal.pcbi.1005595, PMID: 28594827 PMC5481147

[ref93] Wickham H. (2016). ggplot2: Elegant Graphics for Data Analysis. New York: Springer-Verlag. Available at: https://ggplot2.tidyverse.org.

[ref94] Wilkins D. (2020). gggenes: draw gene arrow maps in “ggplot2”. Available at: https://CRAN.R-project.org/package=gggenes (Accessed May 30, 2022).

[ref95] Wuyts S. Wittouck S. De Boeck I. Allonsius C. N. Pasolli E. Segata N.. (2017). Large-scale phylogenomics of the *lactobacillus casei* group highlights taxonomic inconsistencies and reveals novel clade-associated features. mSystems 2, e00061–e00017. doi: 10.1128/mSystems.00061-17 28845461 PMC5566788

[ref96] Ye J. Coulouris G. Zaretskaya I. Cutcutache I. Rozen S. Madden T. L. (2012). Primer-BLAST: a tool to design target-specific primers for polymerase chain reaction. BMC Bioinformatics 13:134. doi: 10.1186/1471-2105-13-134 22708584 PMC3412702

[ref97] Yu G. Smith D. K. Zhu H. Guan Y. Lam T. T.-Y. (2017). Ggtree: an R package for visualization and annotation of phylogenetic trees with their covariates and other associated data. Methods Ecol. Evol. 8, 28–36. doi: 10.1111/2041-210X.12628

[ref98] Zhou L. Yu G. (2021). Ggmsa:a visual exploration tool for multiple sequence alignment and associated data. Available at: http://yulab-smu.top/ggmsa/ (Accessed March 3, 2022). 10.1093/bib/bbac222 35671504

[ref99] Zhou Y. Liang Y. Lynch K. H. Dennis J. J. Wishart D. S. (2011). PHAST: a fast phage search tool. Nucleic Acids Res. 39, W347–W352. doi: 10.1093/nar/gkr485, PMID: 21672955 PMC3125810

