# Corrigendum: New perspectives on an old grouping: the genomic and phenotypic variability of *Oxalobacter formigenes* and the implications for calcium oxalate stone prevention

**DOI:** 10.3389/fmicb.2023.1231746

**Published:** 2023-06-12

**Authors:** John A. Chmiel, Charles Carr, Gerrit A. Stuivenberg, Robertson Venema, Ryan M. Chanyi, Kait F. Al, Daniel Giguere, Henry Say, Polycronis P. Akouris, Sergio Ari Domínguez Romero, Aaron Kwong, Vera Tai, Susan F. Koval, Hassan Razvi, Jennifer Bjazevic, Jeremy P. Burton

**Affiliations:** ^1^Department of Microbiology and Immunology, Western University, London, ON, Canada; ^2^Canadian Centre for Human Microbiome and Probiotics Research, London, ON, Canada; ^3^Department of Molecular Genetics, University of Toronto, Toronto, ON, Canada; ^4^Department of Medicine, The University of British Columbia, Vancouver, BC, Canada; ^5^School of Veterinary Science, Massey University, Palmerston North, New Zealand; ^6^Department of Biology, Western University, London, ON, Canada; ^7^Department of Medicine, Western University, London, ON, Canada; ^8^Division of Urology, Department of Surgery, Western University, London, ON, Canada

**Keywords:** oxalate degradation, *Oxalobacter formigenes*, kidney stone disease, gut microbiome, revised taxonomy, phylogenomic and comparative genomic analyses, nephrolithiasis, hyperoxaluria

In the published article, there was an error in the protologues in which the newly proposed species *Oxalobacter aliiformigenes* sp. nov, *Oxalobacter paeniformigenes* sp. nov, and *Oxalobacter paraformigenes* sp. nov. that were described omitted the information on the culture collection accession identifiers for the three respective type strains. These identifiers are now available and so, to comply with Rule 27(3) of the International Code of Nomenclature of Prokaryotes (Oren et al., 2023), these three protologues are reprinted here in full.

A correction has been made to **Taxonomic and Nomenclature Proposals**, Description of *Oxalobacter aliiformigenes* sp. nov. The section previously stated:

*Oxalobacter aliiformigenes* (a.li.i.for.mi'ge.nes. L. masc. adj. *alius*, other; N.L. part. adj. *formigenes*, formic acid producing, and specific epithet of an *Oxalobacter* species; N.L. part. adj. *aliiformigenes*, meaning that this species is related to but distinct from *Oxalobacter formigenes*).

Cells are Gram-stain negative, rod-shaped with rounded ends typically measuring 1.6 – 2.1 × 0.8 – 1.1 μm on average (estimated from Gram stain), and occurring in singles, pairs, or sometimes in chains. Cells occasionally present as curved. Endospores not found. Flagella not detected. Anaerobic, but aerotolerant with chemotrophic metabolism. Oxalate is useda a major carbon and energy source but typically slow growing in oxalate broth. Optimal growth is at 37°C. Indole not formed. Does not appear to reduce nitrate or sulfate. High levels of the fatty acid C19:0 cyclopropane are present and diagnostically useful (Allison et al., 1985). Members of *O. aliiformigenes* can be distinguished from other species in the genus *Oxalobacter* based on phylogenetic analysis and overall genome relatedness indices. Average genome size ranges from 2.2 – 2.4 Mbp and G+C content of the DNA ranges from 50.9 – 51.5%.

The type strain Va3^T^ (=ATCC XXXX =DSM XXXX) was isolated from a human fecal sample (Duncan et al., 2002).

The corrected section appears below:

*Oxalobacter aliiformigenes* (a.li.i.for.mi'ge.nes. L. masc. adj. *alius*, other; N.L. part. adj. *formigenes*, formic acid producing, and specific epithet of an *Oxalobacter* species; N.L. part. adj. *aliiformigenes*, meaning that this species is related to but distinct from *Oxalobacter formigenes*).

Cells are Gram-stain negative, rod-shaped with rounded ends typically measuring 1.6 – 2.1 × 0.8 – 1.1 μm on average (estimated from Gram stain), and occurring in singles, pairs, or sometimes in chains. Cells occasionally present as curved. Endospores not found. Flagella not detected. Anaerobic, but aerotolerant with chemotrophic metabolism. Oxalate is used as a major carbon and energy source but can be slow growing in oxalate broth. Optimal growth is at 37°C. Indole not formed. Does not appear to reduce nitrate or sulfate. High levels of the fatty acid C19:0 cyclopropane are present and diagnostically useful (Allison et al., 1985). Members of *O. aliiformigenes* can be distinguished from other species in the genus *Oxalobacter* based on phylogenetic analysis and overall genome relatedness indices. Average genome size ranges from 2.2 to 2.4 Mbp and G+C content of the DNA ranges from 50.9–51.5%.

The type strain Va3^T^ (=ATCC TSD-348 =DSM 115068) was isolated from a human fecal sample (Duncan et al., 2002).

A correction has been made to **Taxonomic and Nomenclature Proposals**, Description of *Oxalobacter paeniformigenes* sp. nov. The section previously stated:

*Oxalobacter paeniformigenes* (pae.ni.for.mi'ge.nes. L. adv. *paene*, almost; N.L. part. adj. *formigenes*, formic acid producing, and specific epithet of an *Oxalobacter* species; N.L. part. adj. *paeniformigenes*, meaning that this species is related to but distinct from *Oxalobacter formigenes*).

Cells are Gram-stain negative, rod-shaped with rounded ends typically measuring 1.4 – 2.2 × 0.8 – 1.2 μm on average (estimated from Gram stain), and occurring in singles, pairs, or sometimes in chains. Cells occasionally present as curved. Endospores not found. Flagella not detected. Anaerobic, but aerotolerant with chemotrophic metabolism. Oxalate is used a major carbon and energy source. Optimal growth is at 37 °C. Indole not formed. Does not appear to reduce nitrate or sulfate. Members of *O. paeniformigenes* can be distinguished from other species in the genus *Oxalobacter* based on phylogenetic analysis and overall genome relatedness indices. The genome size of the type strain is 1.93 Mb and the G+C content is 53.8%.

The type strain OxGP1^T^ (=ATCC XXXX =DSM XXXX) was isolated from guinea pig cecum.

The corrected section appears below:

*Oxalobacter paeniformigenes* (pae.ni.for.mi'ge.nes. L. adv. *paene*, almost; N.L. part. adj. *formigenes*, formic acid producing, and specific epithet of an *Oxalobacter* species; N.L. part. adj. *paeniformigenes*, meaning that this species is related to but distinct from *Oxalobacter formigenes*).

Cells are Gram-stain negative, rod-shaped with rounded ends typically measuring 1.4 – 2.2 × 0.8 – 1.2 μm on average (estimated from Gram stain), and occurring in singles, pairs, or sometimes in chains. Cells occasionally present as curved. Endospores not found. Flagella not detected. Anaerobic, but aerotolerant with chemotrophic metabolism. Oxalate is used as a major carbon and energy source. Optimal growth is at 37°C. Indole not formed. Does not appear to reduce nitrate or sulfate. Members of *O. paeniformigenes* can be distinguished from other species in the genus *Oxalobacter* based on phylogenetic analysis and overall genome relatedness indices. The genome size of the type strain is 1.93 Mb and the G+C content is 53.8%.

The type strain OxGP1^T^ (=ATCC TSD-347=DSM 115066) was isolated from guinea pig cecum.

A correction has been made to **Taxonomic and Nomenclature Proposals**, Description of *Oxalobacter paraformigenes* sp. nov. The section previously stated:

*Oxalobacter paraformigenes* (pa.ra.for.mi'ge.nes. Gr. pref. *para*, beside; N.L. part. adj. *formigenes*, formic acid producing, and specific epithet of an *Oxalobacter* species; N.L. part. adj. *paraformigenes*, meaning that this species is related to but distinct from *Oxalobacter formigenes*).

Cells are Gram-stain negative, rod-shaped with rounded ends typically measuring 1.6 – 2.4 × 0.8 – 1.1 μm on average (estimated from Gram stain), and occurring in singles, pairs, or sometimes in chains. Cells occasionally present as curved. Endospores not found. Flagella not detected. Anaerobic, but aerotolerant with chemotrophic metabolism. Oxalate is used a major carbon and energy source. Optimal growth is at 37°C. Indole not formed. Does not appear to reduce nitrate or sulfate. Members of *O. paraformigenes* can be distinguished from other species in the *Oxalobacter* genus based on phylogenetic analysis and overall genome relatedness indices. The genome size of the type strain is 2.49 Mb and the G+C content is 52.7%.

The type strain *O. paraformigenes* HOxBLS^T^ (=ATCC XXXX =DSM XXXX) was isolated from human fecal material.

The corrected section appears below:

*Oxalobacter paraformigenes* (pa.ra.for.mi'ge.nes. Gr. pref. *para*, beside; N.L. part. adj. *formigenes*, formic acid producing, and specific epithet of an *Oxalobacter* species; N.L. part. adj. *paraformigenes*, meaning that this species is related to but distinct from *Oxalobacter formigenes*).

Cells are Gram-stain negative, rod-shaped with rounded ends typically measuring 1.6 – 2.4 × 0.8 – 1.1 μm on average (estimated from Gram stain), and occurring in singles, pairs, or sometimes in chains. Cells occasionally present as curved. Endospores not found. Flagella not detected. Anaerobic, but aerotolerant with chemotrophic metabolism. Oxalate is used as a major carbon and energy source. Optimal growth is at 37°C. Indole not formed. Does not appear to reduce nitrate or sulfate. Members of *O. paraformigenes* can be distinguished from other species in the *Oxalobacter* genus based on phylogenetic analysis and overall genome relatedness indices. The genome size of the type strain is 2.49 Mb and the G+C content is 52.7%.

The type strain *O. paraformigenes* HOxBLS^T^ (=ATCC TSD-346=DSM 115067) was isolated from human fecal material.

The authors apologize for this error and state that this does not change the scientific conclusions of the article in any way.
