# Supplementary material for: New perspectives on an old grouping: The genomic and phenotypic variability of Oxalobacter formigenes and the implications for calcium oxalate stone prevention
Source: Front Microbiol. 2022 Dec 21;13:1011102. doi: 10.3389/fmicb.2022.1011102 (PMC9812493; doi:10.3389/fmicb.2022.1011102)
Supplement: Supplementary file 6 [file Data_Sheet_1.docx]

Supplementary Material


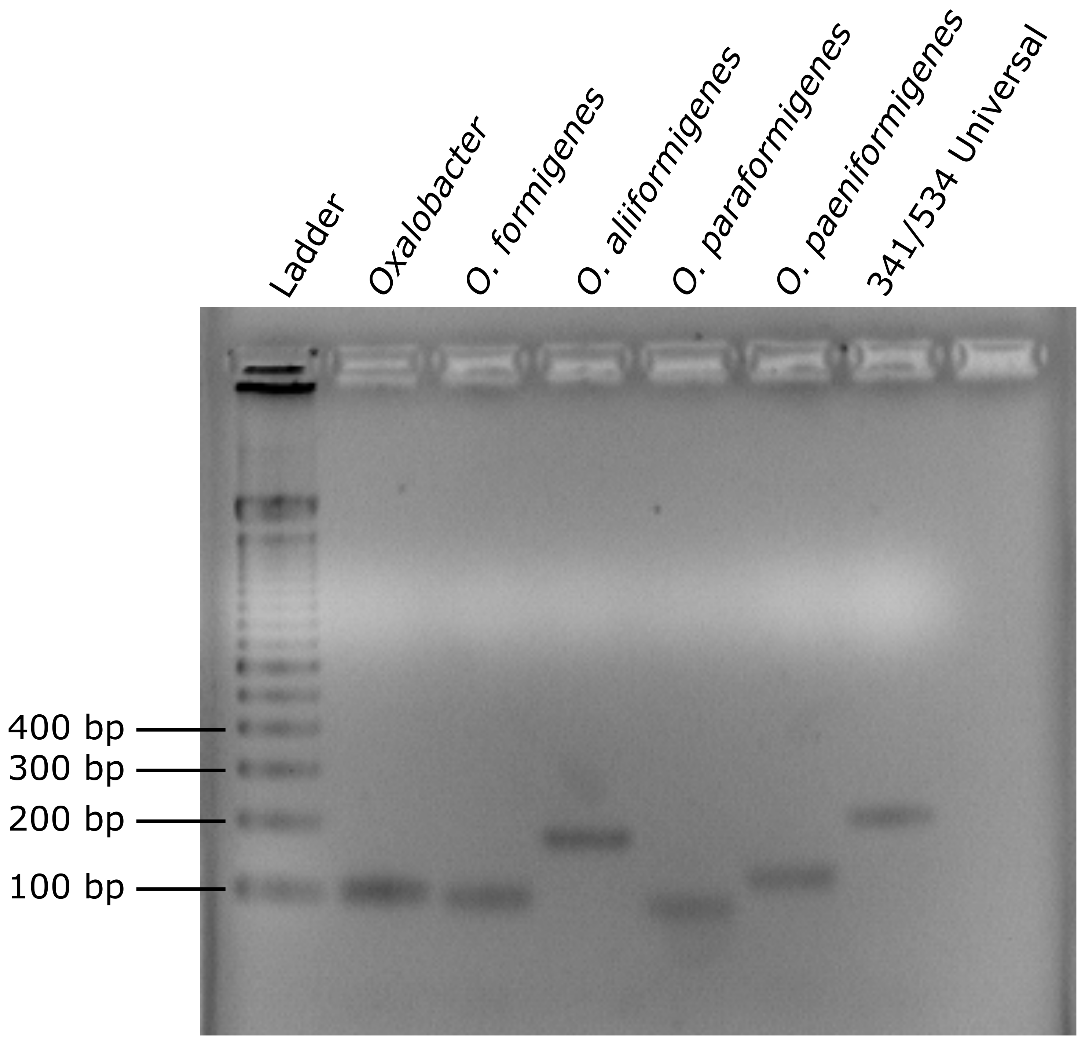


**Supplementary Figure 1.** Primers shown are for *Oxalobacter* (genus specific, but not *O. vibrioformis*), *O. formigenes*, *O. aliiformigenes*, *O. paraformigenes*, *O. paeniformigenes*, and 341/534 universal bacterial primer.


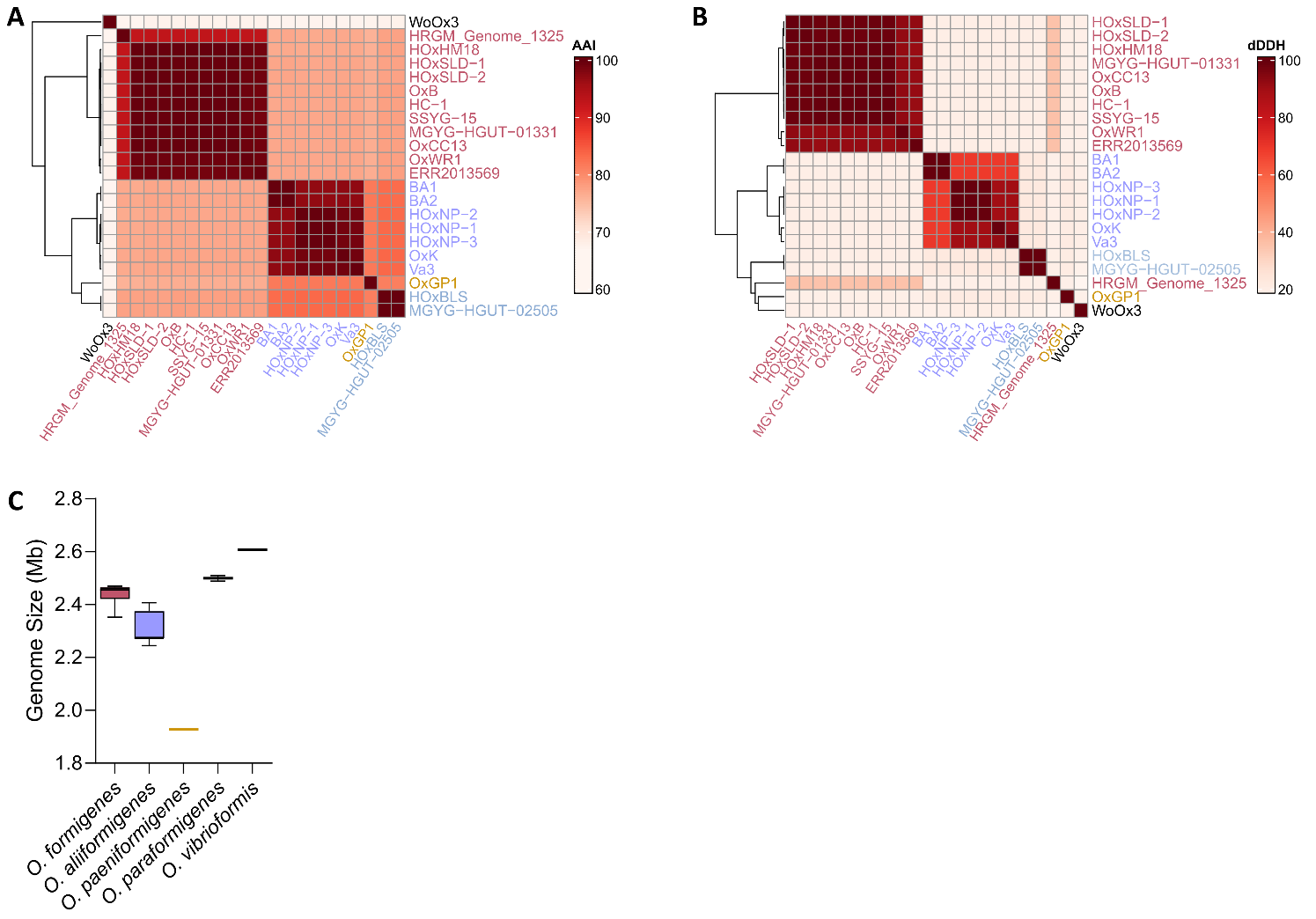


**Supplemental Figure 2.** **(A)** Heatmap depicting average amino acid identity (AAI) pairwise comparisons of strains used in this study, including *O. vibrioformis* WoOx3. **(B)** Heatmap depicting digital DNA-DNA hybridization (dDDH) values of strains used in this study. **(C)** Box and whisker plot displays the whole genome percent GC content of each of the *Oxalobacter* species in this study. Boxes represent first and third quartile values while black line denoting the median, and whiskers encompass maximum and minimum values.


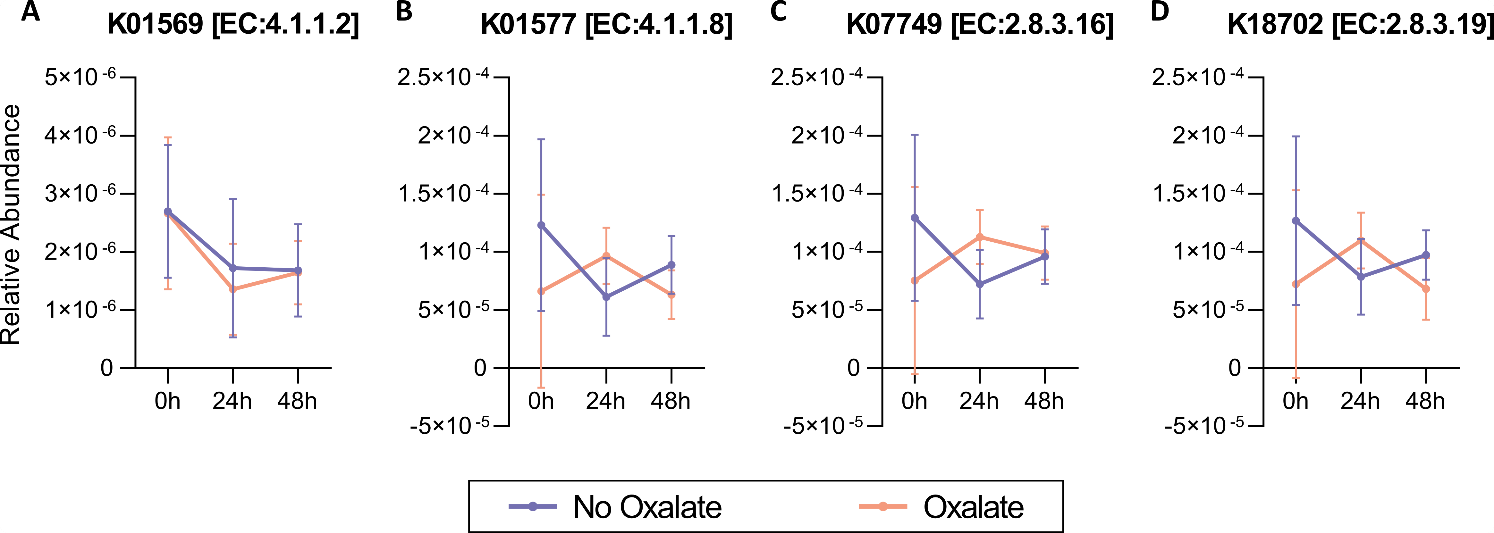


**Supplemental Figure 3**. **(A ­– D)** Relative abundance of predicted oxalate degrading KEGG Orthology numbers over time. Data are displayed as mean ± SD.


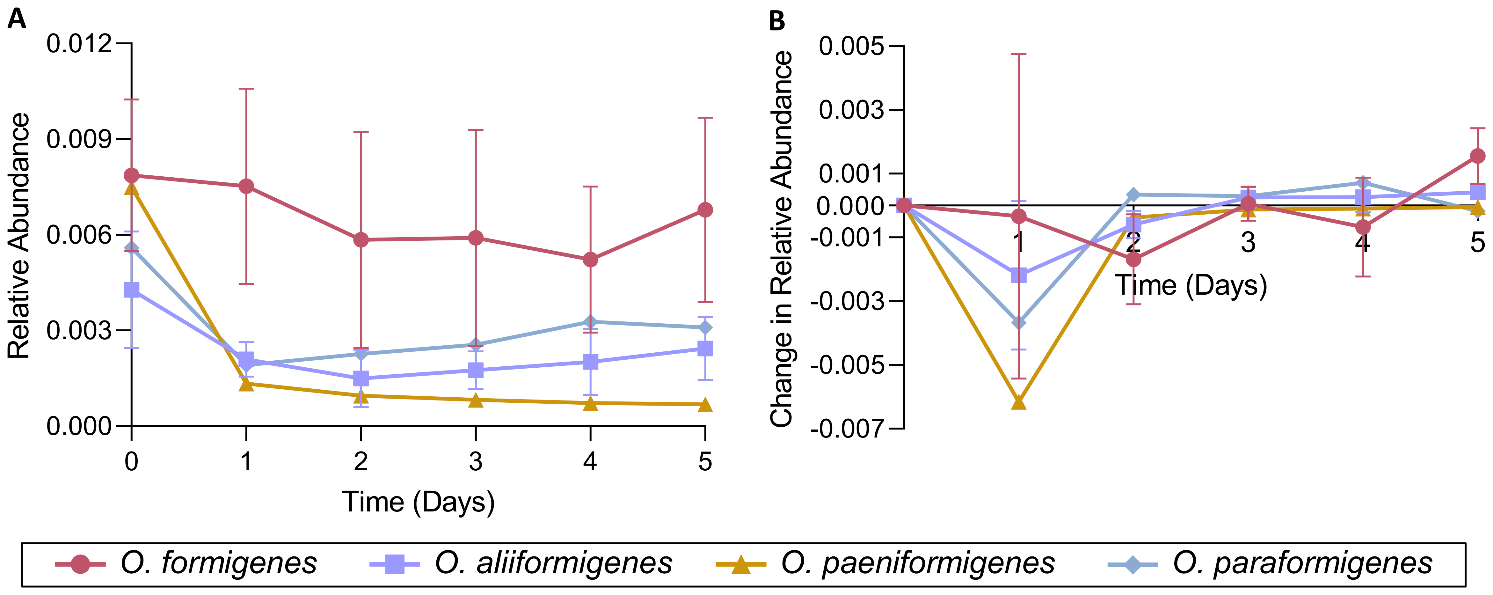


Supplemental Figure 4. (A) Relative abundance and (B) change in relative abundance of each strain over time as detected by genus-specific qPCR analysis. Data are displayed as mean ± SD.
